# MiR-211 determines brain metastasis specificity through SOX11/NGN2 axis in triple-negative breast cancer

**DOI:** 10.1038/s41388-021-01654-3

**Published:** 2021-02-03

**Authors:** Jhih-Kai Pan, Cheng-Han Lin, Yao-Lung Kuo, Luo-Ping Ger, Hui-Chuan Cheng, Yun-Chin Yao, Michael Hsiao, Pei-Jung Lu

**Affiliations:** 1 Institute of Clinical Medicine, College of Medicine, National Cheng Kung University, Tainan, Taiwan; 2 Department of General Surgery, National Cheng Kung University Hospital, Tainan, Taiwan; 3 Department of Medical Education and Research, Kaohsiung Veterans General Hospital, Kaohsiung, Taiwan; 4 Clinical Medicine Research Center, National Cheng Kung University Hospital, Tainan, Taiwan; 5 Genomics Research Center, Academia Sinica, Taipei, Taiwan; 6 Department of Biochemistry, College of Medicine, Kaohsiung Medical University, Kaohsiung, Taiwan; 7 Department of Clinical Medicine Research, National Cheng Kung University Hospital, Tainan, Taiwan

**Keywords:** Breast cancer, Metastasis

## Abstract

Brian metastasis, which is diagnosed in 30% of triple-negative breast cancer (TNBC) patients with metastasis, causes poor survival outcomes. Growing evidence has characterized miRNAs involving in breast cancer brain metastasis; however, currently, there is a lack of prognostic plasma-based indicator for brain metastasis. In this study, high level of miR-211 can act as brain metastatic prognostic marker in vivo. High miR-211 drives early and specific brain colonization through enhancing trans-blood–brain barrier (BBB) migration, BBB adherence, and stemness properties of tumor cells and causes poor survival in vivo. SOX11 and NGN2 are the downstream targets of miR-211 and negatively regulate miR-211-mediated TNBC brain metastasis in vitro and in vivo. Most importantly, high miR-211 is correlated with poor survival and brain metastasis in TNBC patients. Our findings suggest that miR-211 may be used as an indicator for TNBC brain metastasis.

## Introduction

Brain metastases are the most common brain malignancy that frequently arises from 15 to 25% advanced breast cancer (BC) patients [1, 2]. The poor survival of patients with brain metastasis is due to limiting therapeutic strategies and lack of prognostic biomarkers. Therefore, identification and characterization of the brain metastasis prognostic biomarker is the critical issue for patients’ treatment. Tumor cells colonizing in brain parenchyma must penetrate the blood–brain barrier (BBB) [1, 3, 4], which protects the brain from infection and toxic substances; the BBB is composed of endothelial cells contacting the tight junction with pericytes and astrocytes [5, 6]. Currently, increasing evidence has demonstrated that tumor cells cause the dysfunction of the BBB to form the blood–tumor barrier, which changes the molecular permeability and leads to the loss of the integrity of the BBB [7–9]. Many additional factors have been identified as potential site-specific mediators of BBB transmigration and perivascular growth, including cathepsin S [4], c-Met [10], Angiopoietin-2 [11], PTGS2 [12], HB-EGF [13], ST6GALNAC5 [14], PLEKHA5 [15], and the serum factor PLGF [16]. Thus, cancer cells interacted with the BBB structure play a critical role in brain metastasis and tumor relapse.

MicroRNAs (miRNAs) are short noncoding RNAs that bind to the 3′-untranslated regions (3′-UTRs) of target genes [17]. Evidence showed that miRNAs are deregulated in various cancer types [18–22] and circulating miRNAs are present and remarkably stable plasma-based biomarkers in human plasma [23]. We hypothesized that miRNAs in plasma/primary tumor tissues may act as prognostic marker to predict brain metastasis in TNBC patients. Previously, upregulation of miR-141 promotes the mesenchymal to epithelial transition in brain-tropic TNBC cells [24]. Overexpression of miR-7 decreases the brain metastatic burden in vivo [25] and upregulation of miR-1258 suppresses BC brain metastasis through targeting heparanase in vivo [26]. However, the underlying mechanism by which miRNAs regulate BBB adherence and trans-BBB migration in brain metastasis remains unclear.

In our study, we established available experimental in vitro and in vivo models of TNBC brain metastasis to investigate and characterize the functional role of miR-211 in TNBC brain metastasis. Plasma high miR-211 was also demonstrated to predict TNBC brain metastasis in vivo.

## Results

### High expression of miR-211 enhances brain metastasis of TNBC

**Fig. 1:**
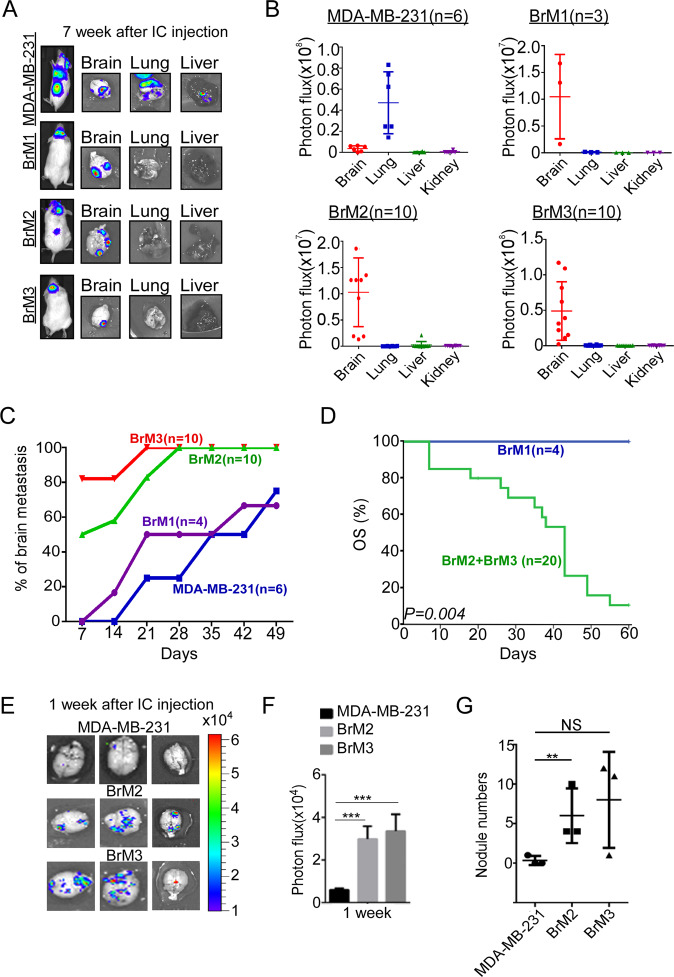
Isolated brain-tropic cells promote early brain metastasis and cause poor survival in vivo. **A** Isolated brain-tropic cells (BrM1, BrM2, and BrM3 cells) and MDA-MB-231 cells were introduced into mice through IC injection. **B** After 7–10 weeks of tumor injection, the organs were collected and photon flux was calculated by IVIS quantitative analysis. **C** Brain metastasis ability of BrM cells was examined by calculating brain metastasis percentage. **D** Kaplan–Meier analysis was used to investigate the overall survival in the xenograft mice model. **E** Brain metastasis was detected using IVIS system 1 week after tumor cell injection in NOD/SCID female mice ex vivo. **F** After 1 week of tumor cell injection, the brain was collected, and photon flux was calculated using IVIS quantitative analysis. **G** The numbers of nodule in the brain was quantified through IVIS quantitative analysis (**P < 0.01; ***P < 0.001; NS no significant difference).

To identify the novel miRNAs involved in brain-specific metastasis in TNBC, brain-tropic BC cell lines (BrM1, BrM2, and BrM3) derived from MDA-MB-231 were established through a series of in vivo selections (Supplementary Fig. S1A, as described in Supplementary information). IC injection followed by IVIS was used to monitor distant metastasis in vivo. Figure 1A, B shows that compared with parental cells that were mainly colonized in the lung, brain-tropic cells increased specificity of brain colonization. Early brain metastasis of mice injected with BrM2 and BrM3 cells could be detected 3–7 days after injection, whereas the parental cell signal was undetectable till the third week after injection (Fig. 1C). Compared with the BrM1 group, the survival rate of mice injected with BrM2 and BrM3 cells was significantly decreased (*P* = 0.004) (Fig. 1D). To further investigate the brain colonization ability of circulating brain-tropic cells, after IC injection for a week, the brain tissues of mice were collected and examined using IVIS for detecting the colonization signals ex vivo. The photon flux, which represents the number of colonization cells, was increased fourfold in the brain-tropic group (BrM2 and BrM3 cells) compared with the parental group (Fig. 1E,F, parental group: 0.21 ± 0.04, BrM2: 3.20 ± 0.74, *P* < 0.001, BrM3: 3.60 ± 0.79, *P* < 0.001). The nodule numbers were also increased in the brain-tropic cells group (Fig. 1G, parental group: 0.3 ± 0.6, BrM2: 6 ± 3.5, *P* = 0.049, BrM3: 8 ± 6.1, *P* = 0.095, unpaired *t*-test). The cell viability rate was analyzed by MTT and orthotopic injection in vitro and in vivo, respectively. The results showed that brain-tropic cells had slower growth ability compared with MDA-MB-231 (Supplementary Fig. S1B, C); however, the anchorage independent growth ability significantly increased in the BrM3 compared with MDA-MB-231 in vitro (Supplementary Fig. S1D). Taken together, our data indicated that isolated brain-tropic cells may cause early brain metastasis and poor survival in vivo.

**Fig. 2:**
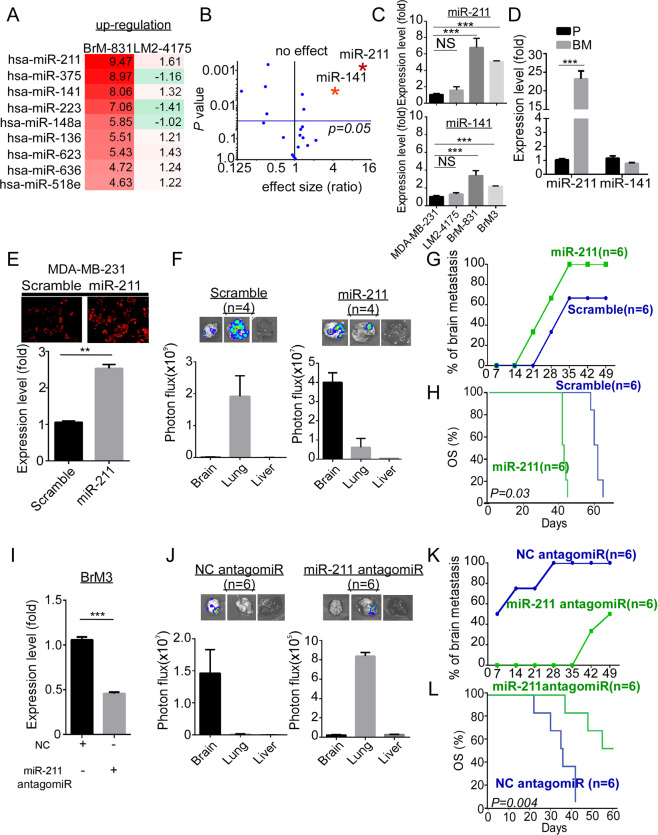
High miR-211 promotes early brain colonization and causes poor survival in vivo. **A** The heat map indicates up- or downregulated miRNAs in BrM-831 or LM2-4175 cells compared with MDA-MB-231 cells. **B** A volcano plot assay compared the expression of miRNAs in BrM-831 cells relative to MDA-MB-231 cells. *P* values (*y*-axis) and effect size (ratio, *x*-axis) were calculated using GraphPad (miR-211 and miR-141 are indicated as red stars). **C** The expression of miR-211 and miR-141 was validated using qRT-PCR in organ-tropic cells. **D** 1 × 10^5^ MDA-MB-231 cells were injected into the mammary gland of female mice. In approximately 5 weeks, the primary tumors were surgically removed, following metastatic brain lesions were collected at 15–20 weeks after orthotopic injection. Primary and metastatic lesions were detected by the IVIS system ex vivo to confirm the tumor lesions. qRT-PCR was used to evaluate the expression of miR-211 and miR-141 in primary tumor and brain metastatic tissues (P primary site, BM brain metastatic site). Three individual primary lesions and three individual brain metastatic lesions were used in the experiment. **E** Using the transfection methods and flow cytometry, scramble, miR-211 stably expressing cell was established in MDA-MB-231 cells. qRT-PCR was used to evaluate the expression of miR-211 in miR-211 stably expressing cell, respectively. **F** MDA-MB-231 and miR-211-overexpressing cells were introduced into the mice through IC injection. After 7–10 weeks, the photon flux of the organs was calculated using IVIS quantitative analysis ex vivo. **G** Brain metastatic ability of MDA-MB-231 cells and miR-211-overexpressing cells was evaluated by calculating the percentage of mice with brain metastases. **H** Kaplan–Meier analysis revealed the overall survival in xenograft mice. **I** miR-211 knockdown cells were established by transiently transfecting NC antagomiR or miR-211 antagomiR in BrM3 cells. The expression of miR-211 in miR-211-suppressing cells was investigated using qRT-PCR. **J** BrM3 and miR-211-suppressing cells were introduced into the mice through IC injection. After 7–10 weeks, the photon flux of organs was calculated using IVIS quantitative analysis ex vivo. **K** Brain metastatic ability of BrM3 cells and miR-211-suppressing cells was investigated by calculating the percentage of mice with brain metastasis. **L** Kaplan–Meier analysis revealed the overall survival in miR-211-suppressing xenograft mice (**P < 0.01; ***P < 0.001; NS no significant difference).

miRNA arrays of lung-tropic (LM2-4175) and brain-tropic (BrM-831) BC cells were performed and compared to reveal potential miRNAs specifically involved in brain metastasis (as described in Supplementary Methods). The differential expression miRNA profiles were ranked based on the three filtering criteria: (1) *P* < 0.01, (2) fold change higher or lower than fourfold compared with control, and (3) Venn diagram excluded the lung metastasis-associated miRNAs (Fig. 2A and Supplementary Fig. S2A). The expression of miR-211 and miR-141 was significantly increased (10.2- and 4.3-fold, respectively) in BrM-831 cells compared with parental cells, as observed in volcano plot analysis (Fig. 2B). The levels of candidate miRNAs in BrM cells were validated by quantitative real-time polymerase chain reaction (qRT-PCR) (Supplementary Fig. S2B). Moreover, Fig. 2C shows that miR-211 and miR-141 significantly increased 4.9-fold (*P* < 0.001) and 2.3-fold (*P* < 0.001), respectively, in BrM3 cells compared with MDA-MB-231 cells. Results also showed that miR-211 significantly increased 24.4-fold (*P* < 0.001) in brain metastatic sites compared with the primary sites, whereas no quantitative change in miR-141 expression was detected in primary sites and brain metastatic sites (Fig. 2D). Taken together, these results suggested that the miR-211 highly expressed in brain metastatic tumors in vitro and in vivo.

To characterize the functional roles of miR-211 in brain metastasis of TNBC, miRNA precursors and antagomiRs were used to overexpress or knockdown miR-211 in MDA-MB-231 and BrM3 cells. The qRT-PCR results showed that miR-211 level significantly increased (2.5-fold, *P* = 0.003) in miR-211-overexpressing MDA-MB-231 cells (Fig. 2E). MiR-211 overexpressing promoted cell invasion and inhibited tumor growth in vitro and in vivo (Supplementary Fig. S3). After IC injection for 7 weeks, the brain tissues were collected and examined using the IVIS to detect the colonization signals ex vivo. Our results showed that compared with the lung and liver, significant brain colony numbers with a high photon flux intensity could be detected in the miR-211-overexpressing group, whereas the scramble group mainly had lung colonization (Fig. 2F). Three weeks after IC injection, 33.3% of mice in the miR-211-overexpressing group had brain metastasis, whereas those in the scramble group showed brain metastasis until to the fourth week after injection. Seven weeks after IC injection, which defined as endpoint, 100% of mice in miR-211-overexpresing group had brain colonization, and 66.7% of mice in scramble group had brain colonization (Fig. 2G). Figure 2H shows that mice in the miR-211-overexpressing group had poorer median overall survival (OS) time (41 days) than that of mice in the scramble group (60 days) (*P* = 0.03). To downregulate miR-211 in BrM3 cells, anti-miR-211 antagomiRs were transiently transfected into BrM3 cells. The knockdown efficiency was evaluated daily by qRT-PCR (Supplementary Fig. S2C). Two days after anti-miR-211 antagomiRs treatment showed significantly downregulated (approximately 50%) miR-211 levels (Fig. 2I, *P* < 0.001). Figure 2J shows that loss of brain-tropic metastasis of BrM3 cells was observed in the miR-211-antagomiR treatment group compared with the NC antagomiR group. Brain metastasis was first detected at 1 week in BrM3 cells-injected mice; however, it was undetected until the sixth week in mice with miR-211-knockdown group (Fig. 2K). Figure 2L shows that mice in miR-211-knockdown group significantly prolonged the median survival time from 38 to 62 days (*P* = 0.004). Taken together, our data suggest that miR-211 specifically promotes early brain colonization and causes poor survival in vivo.

### High miR-211 in plasma can act as TNBC brain metastatic indicator and is correlated with brain metastasis in TNBC patients

**Fig. 3:**
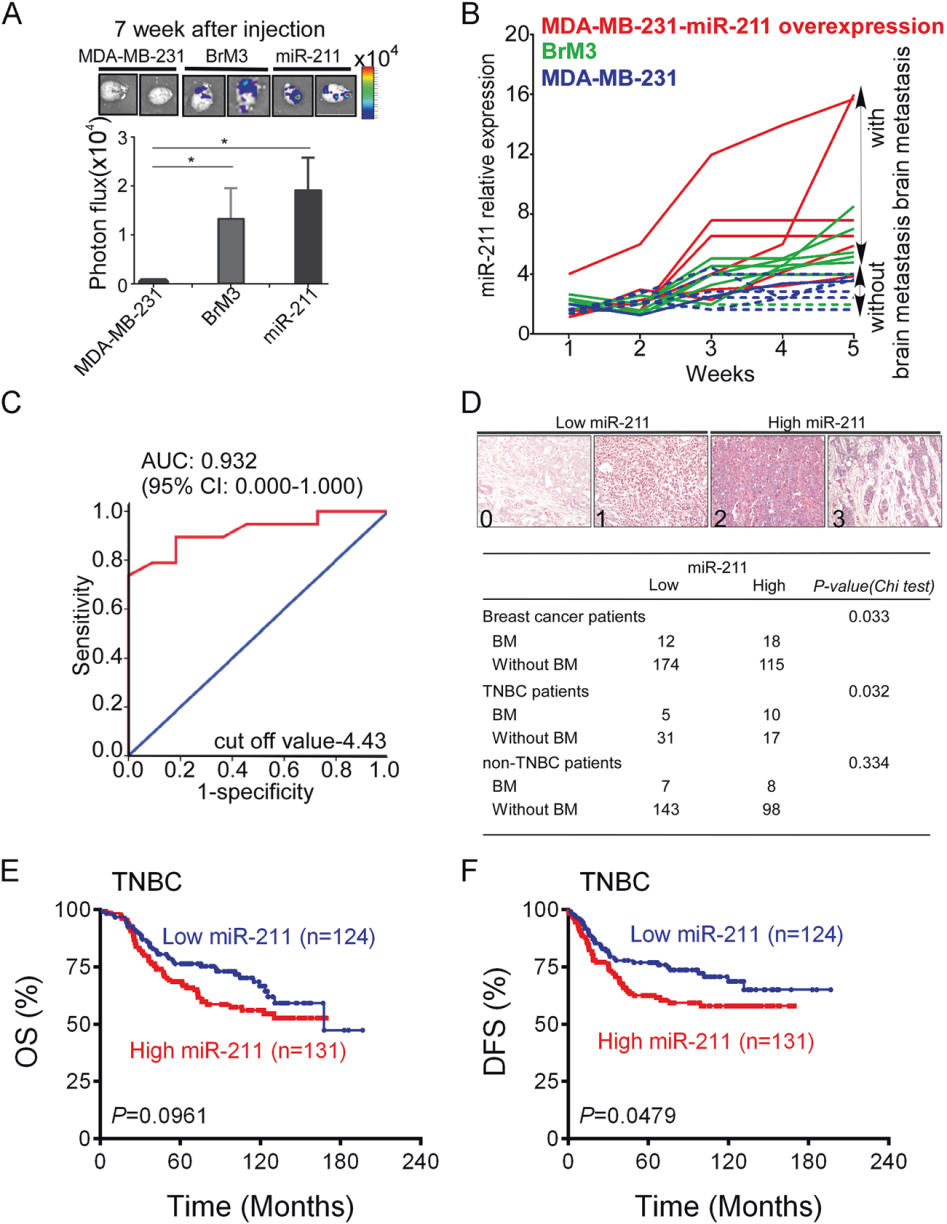
High miR-211 acts as prognostic marker for brain metastasis in vivo and is correlated with brain metastasis and poor survival in BC patients. **A** Brains were collected and detected using IVIS quantitative intensity analysis ex vivo. **B** The level of circulating miR-211 in mice plasma was evaluated using qRT-PCR once a week. **C** ROC curve was plotted to evaluate the prognostic power of circulating miR-211 expression for brain metastasis in xenograft mice. **D** miR-211 expression was examined by ISH in 319 tissues (included 63 samples from TNBC patients and 256 non-TNBC patients; 30 patients with brain metastasis and 289 with metastasis to other sites), and the expression of miR-211 was scored at four different levels (0,1=low miR-211 expression; 2,3=high miR-211 expression). **E** Overall survival. **F** Disease-free survival of the 255 TNBC patients was analyzed by Kaplan–Meier analysis after stratification by miR-211 expression level (*P < 0.05).

Tumor-derived circulating RNAs have been detected in whole plasma, and they help develop indicators that enable the detection of metastasis in tumors. qRT-PCR analysis was used to investigate plasma miR-211 in NOD-SCID mice orthotopically inoculated with MDA-MB-231 cells, miR-211-overexpressing cells, and BrM3 cells. Brain metastasis was detected through IVIS in the miR-211-overexpressing group and BrM3 group 7 weeks after tumor cells were inoculated. Significantly increased tumor cells were observed in the brains of mice in the miR-211-overexpressing (*P* = 0.048) and BrM3 (*P* = 0.044) groups (Fig. 3A). qRT-PCR was performed every 7 days to evaluate the changes in the miR-211 level in the mice plasma during the first 5 weeks, and the endpoint of brain metastasis experiment was set at 10 weeks after inoculation of the tumor cells. To evaluate the indicator role of miR-211 to predict brain metastasis in vivo, the plasma miR-211 of mice were detected by qRT-PCR. The mean value was used as cut-off value to determine the high and low level of miR-211 in plasma. According to our results showed in Fig. 3B, mice with plasma miR-211 higher than cut-off value had brain metastasis (solid line). Mice with plasma miR-211 lower than cut-off value did not have brain metastasis (dashed line). The result indicated that the high plasma miR-211 level was associated with brain metastasis. The receiver operating characteristic (ROC) curve of miR-211 was then generated and analyzed to predict brain metastasis with a cut-off value of 4.43 and the corresponding area under the ROC curve (AUC) of 0.932 (95% CI: 0.000–1.000) (Fig. 3C). According to these results, we suggest that high miR-211 expression level in plasma acts as a prognostic indicator for brain metastasis. To further clarify the brain metastasis indicative role of miR-211 in BC patients, tissue arrays with 484 BC patients (255 TNBC patients and 229 non-TNBC patients, Supplementary Fig. S4A and Supplementary Table S1) were used to detect miR-211 expression by ISH. There were 319 patients with metastasis in our cohorts, 30 patients with brain metastasis (15 TNBC patients, and 15 non-TNBC patients). ISH of miR-211 showed that the high miR-211 was significantly correlated with brain metastasis in BC and TNBC patients (*P* = 0.033 and *P* = 0.032, respectively, *χ*^2^ analysis), whereas it showed no significant difference in non-TNBC patients (*P* = 0.334) (Fig. 3D). Kaplan–Meier analysis was then used to evaluate the prognostic role of miR-211. The results indicated that high miR-211 was correlated with poor OS (*P* = 0.043) and poor disease-free survival (DFS, *P* = 0.0286) (Supplementary Fig. S4B). The association of miR-211 expression with OS and DFS was further analyzed in TNBC and non-TNBC patients by Kaplan–Meier analysis. The results indicated that high miR-211 was correlated with TNBC DFS (*P* = 0.0479, Fig. 3F) but not correlated with TNBC OS (*P* = 0.0961, Fig. 3E), non-TNBC OS (*P* = 0.4281), and DFS (*P* = 0.2257) (Supplementary Fig. S4B). The OS and DFS of different subtypes of non-TNBC patients were also analyzed and results showed that no significant correlation was observed (Supplementary Fig. S4B). The other two independent cohorts also supported that the high circulating miR-211 was significantly correlated with poor survival in patients with BC (*P* = 0.034) (Supplementary Fig. S4C). In the TCGA database, the high miR-211 levels were correlated with poor survival in 1262 BC patients (Supplementary Fig. S4D). Taken together, our data demonstrated that the high miR-211 was a strong prognostic indicator of brain metastasis and was correlated with poor survival.

### High miR-211 enhances tumor cell adhesion and trans-BBB ability to promote brain metastasis

**Fig. 4:**
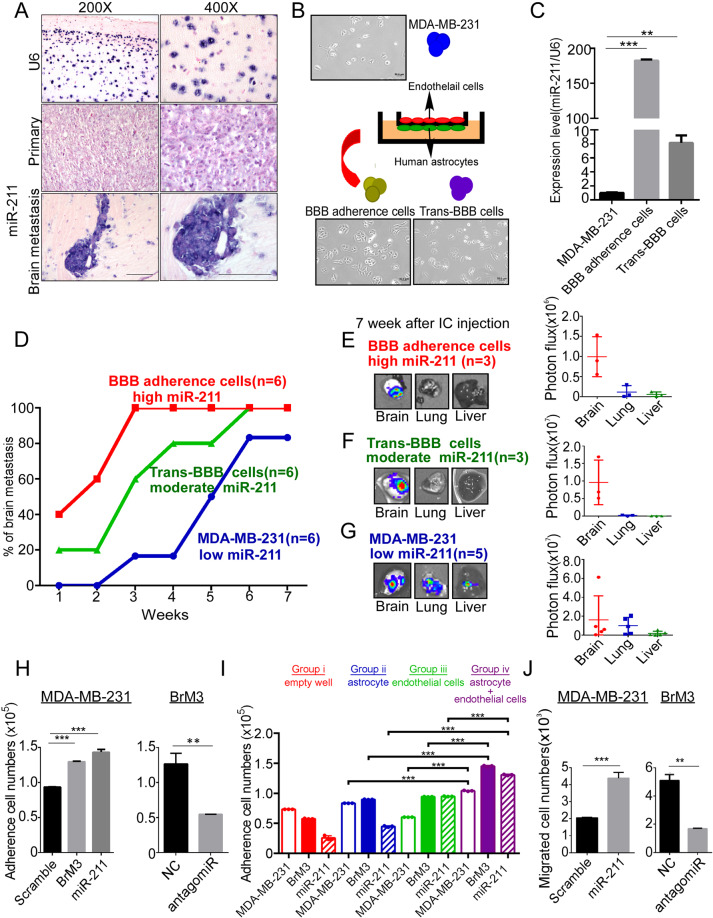
High miR-211 enhances BBB adherence and penetration abilities of TNBC cells to promote early brain metastasis. **A** 1 × 10^5^ BrM1 cells were injected into the mammary gland of female mice. The primary tumor was surgically removed at fifth week, following metastatic brain lesions were collected at 15–20 weeks after orthotopic injection. Primary and metastatic lesions were detected by the IVIS system ex vivo to confirm the tumor lesions. Five individual primary lesions and six individual brain metastatic lesions were used in the experiment. MiR-211 expression was detected by ISH in xenografted tissues. U6 acted as control. Purple color indicated miR-211 expression by nitro blue tetrazolium/5-Bromo-4-chloro-3-indolyl-phosphate (NBT/BCIP) staining, followed by counterstaining with Nuclear Fast Red indicting nuclear staining. **B** In vitro artificial BBB was used to isolate the BBB adherence and trans-BBB cells. **C** qRT-PCR was used to examine the miR-211 in BBB adherence cells and trans-BBB cells compared with MDA-MB-231 cells. **D** Brain metastatic ability of MDA-MB-231 cells, BBB adherence cells, and trans-BBB cells was evaluated by calculating the percentage of mice with brain metastasis. **E**–**G** The photon flux of organs was analyzed using IVIS ex vivo. **H** BBB adherence ability of miR-211-overexpressing or -suppressing cells was investigated using the In vitro artificial BBB. Adhesive cell numbers were counted by the luciferase activity of cells. **I** Luciferase activity detection was used to analyze the number of adherent cells in three culture conditions: (i) empty well, (ii) human astrocytes only, (iii) endothelial cells only, and (iv) a co-culture of human astrocytes and endothelial cells. **J** HUVECs were co-cultured with human astrocytes on trans-well insert for 3 days. MDA-MB-231, BrM3, and miR-211-overexpressing or -suppressing cells were seeded on the upper chamber to evaluate trans-BBB migration in 48 h. The number of migrated cells was counted by luciferase activity of cells (**P < 0.01; ***P < 0.001).

High miR-211 intensity was observed in brain metastatic sites by ISH (Fig. 4A). To investigate whether miR-211 affects tumor cell adhesion and trans-BBB ability, in vitro artificial BBB was established (Fig. 4B). The structural integrity of the artificial BBB was investigated using sodium fluorescence tracking dye to exclude the possibility of tumor cell invasion owing to the compromised structural integrity of the BBB (Supplementary Fig. S5A). The luciferase activities of tumor cells were calculated and represented the cell numbers in vitro (Supplementary Fig. S5B). Most importantly, the result demonstrated that miR-211 levels in BBB adherence and trans-BBB cells significantly increased to 193.3-fold (*P* < 0.001) and 7.2-fold (*P* = 0.003), respectively, compared with parental cells (Fig. 4C). Thus, we concluded that the high miR-211 level enhanced the BBB adherence and trans-BBB migration ability of tumor cells, which led to early brain colonization and brain-tropic ability. The brain metastatic capacity of BBB adherent cells, trans-BBB cells, or parental 231 cells was evaluated in vivo. First, mice injected with cells with high miR-211 levels showed brain metastasis at 1 week, whereas the brain metastasis in the control group was detected at 3 weeks (Fig. 4D). Second, cells with high miR-211 levels showed brain-tropic ability ex vivo (Fig. 4E–G). Third, Fig. 4H shows that the adhesion cell numbers in high miR-211 expressing BrM3 and miR-211-overexpressing groups significantly increased to 1.3 × 10^5^ and 1.5 × 10^5^, respectively, compared with the scramble group (0.9 × 10^5^) in vitro artificial BBB condition (*P* = 0.0003 in BrM3 group; *P* = 0.0004 in miR-211-overexpressing group). By contrast, the adhesion cell numbers significantly decreased from 1.4 × 10^5^ to 0.5 × 10^5^ in miR-211-knockdown BrM3 cells compared with the control group (*P* = 0.0012). The high miR-211 mediating adhesion ability was also evaluated in another TNBC cells, HCC1806. The result showed that adhesion cell numbers in miR-211-overexpressing HCC1806 also significantly increased compared with the scramble group, and the result further supported that high miR-211 expression promoted TNBC cells’ adhesion ability (Supplementary Fig. S5C). Fourth, the adherence ability of cells with high miR-211 was significantly increased in the bilayer of astrocyte and endothelial cell co-culture, which represented the environment of BBB, compared with the monolayer of astrocytes or endothelial cells (Fig. 4I, bilayer: 1.7 × 10^5^ cells in the BrM3 group, 1.4 × 10^5^ cells in the miR-211-overexpressing group; monolayer: 1.0 × 10^5^ cells in the BrM3 group, 1.1 × 10^5^ cells in the miR-211-overexpressing group; *P* = 0.0002 in BrM3 group; *P* = 0.0003 in the miR-211 group). Fifth, our results show that the trans-BBB migration ability was significantly increased in the miR-211-overexpressing group (2.0 × 10^3^ cells in scramble group; 4.6 × 10^3^ cells in the miR-211-overexpressing group, *P* = 0.0003). By contrast, miR-211-knockdown in BrM3 cells significantly reduced their trans-BBB migration ability (1.8 × 10^3^ cells in miR-211-knockdown BrM3 group, 5.2 × 10^3^ cells in the control group, *P* = 0.0032) (Fig. 4J).

### High miR-211 promotes cancer stem cell properties in TNBC

**Fig. 5:**
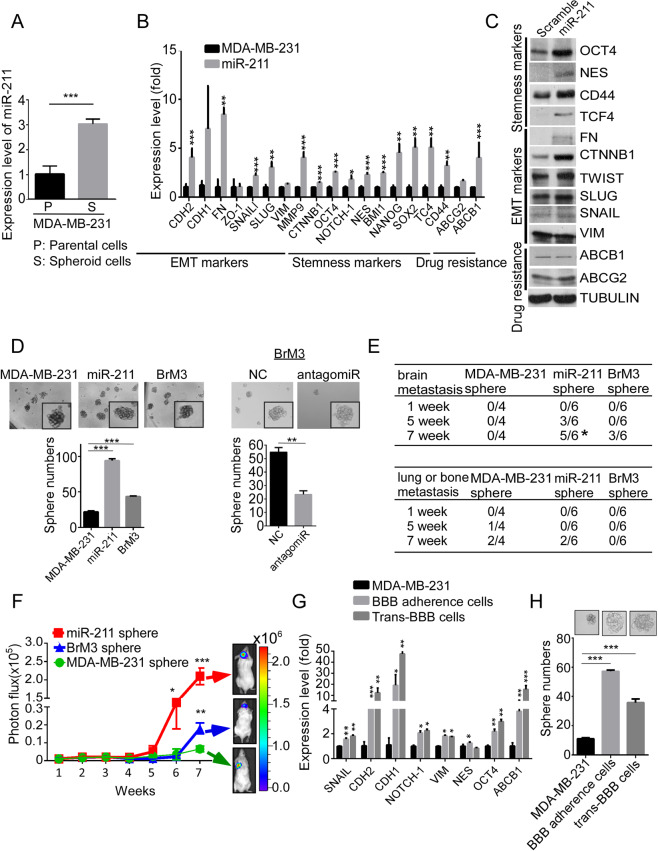
High miR-211 enhances stemness properties of TNBC cells. **A** The expression of miR-211 was evaluated in the parental and spheroid MDA-MB-231 cells by qRT-PCR (P parental cells, S spheroid cells); in the miR-211-overexpressing cells and MDA-MB-231 cells. **B** The expression of epithelial–mesenchymal transition (EMT)-related genes, stemness genes, and drug resistance genes was examined using qRT-PCR. **C** The protein levels of EMT-related genes, stemness genes, and drug resistance genes were examined using western blotting. **D** The sphere-forming abilities of MDA-MB-231, miR-211-overexpressing MDA-MB-231, and BrM3 and miR-211-suppressing BrM3 cells were examined by sphere-formation assay. In total, 1000 MDA-MB-231, miR-211-overexpressing and BrM3 spheroid cells were IC injected in mice. **E** The metastatic ability was monitored using IVIS. **F** The metastatic brain signal was analyzed by using IVIS quantitative analysis. **G** The expression of EMT and stemness genes in BBB adherence and trans-BBB cells was investigated by qRT-PCR. **H** Stemness property was evaluated by sphere-formation assay in vitro (*P < 0.05; **P < 0.01; ***P < 0.001).

To investigate whether the high miR-211 level increased the stemness properties to promote early brain colonization in TNBC cells, the sphere-formation assay was performed and qRT-PCR result shows that the miR-211 level significantly increased in spheroid cells compared with parental cells (Fig. 5A, *P* < 0.0001). Stemness-related genes and proteins were significantly increased in miR-211-overexpressing cells (Fig. 5B, C). Moreover, two-way demonstrations by overexpression and knockdown of miR-211 indicated that miR-211 levels could regulate the stemness ability. First, Fig. 5D shows that sphere numbers were significantly increased in high miR-211-expressing cells compared with control cells (sphere numbers: MDA-MB-231, 23 ± 0.4; miR-211-overexpressing cells, 98 ± 1.7; and BrM3, 47 ± 1.2; *P* < 0.0001 in miR-211 group; *P* < 0.0001 in BrM3 group). Second, Supplementary Fig. S6A, B also shows that sphere numbers were significantly increased in miR-211 overexpressing HCC1806 cells (sphere numbers: 60 ± 3.5 in the HCC1806 group and 114 ± 3.2 in the HCC1806 miR-211-overexpressing group, *P* = 0.0032). Third, the sphere numbers of miR-211-antagomiR treated BrM3 cells were significantly decreased from 55 ± 4.3 to 24 ± 3.8 (*P* = 0.002). Fourth, IVIS showed that high brain metastasis was observed in mice injected with miR-211-overexpressing cells (*P* = 0.0476, Fisher’s exact test) and BrM3 spheres at 7 weeks, whereas those injected with MDA-MB-231 spheres mainly showed lung or bone metastasis (Fig. 5E). Quantitative results indicated that the photon intensity of brain metastasis was significantly increased in mice injected with miR-211-overexpressing and BrM3 cells, whereas the photon intensity of lung metastasis was increased in the control group (Fig. 5F). The stemness-related genes and sphere numbers were also significantly increased in BBB adhesion and trans-BBB cells compared with control cells (Fig. 5G, H; sphere numbers: *P* < 0.0001 in BBB adhesion cells; *P* < 0.0001 in trans-BBB cells), and our in vitro and in vivo results suggested that miR-211-mediated early brain metastasis might be attributed to the increased stemness properties of TNBC cells.

### MiR-211 mediates TNBC brain metastasis by downregulating the SOX11/NGN2-dependent axis

**Fig. 6:**
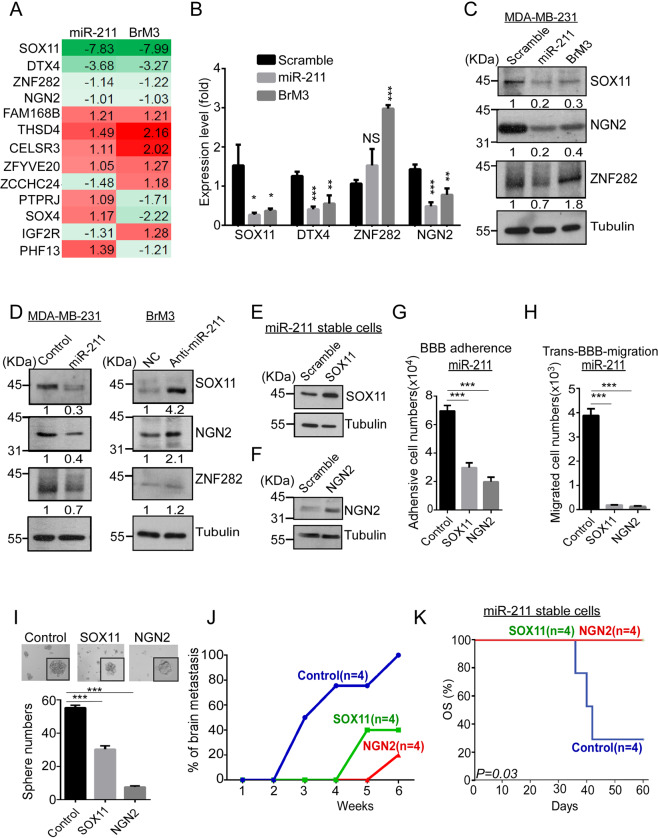
MiR-211 mediates brain metastasis of TNBC through downregulating SOX11/NGN2-dependent axis. **A** Gene expression microarray analysis was used to identify upregulated or downregulated genes in miR-211-overexpressing and BrM3 cells compared with MDA-MB-231 cells. **B** The RNA levels of *SOX11*, *DTX4*, *ZNF282*, and *NGN2* were evaluated by qRT-PCR in miR-211-overexpressing and BrM3 cells compared with control cells. **C** The protein levels of SOX11, ZNF282, and NGN2 were investigated by western blotting in miR-211-overexpressing and BrM3 cells compared with control cells. **D** MDA-MB-231 or BrM3 cells were transfected with miR-211 mimics or miR-211 antagomiR, respectively. Western blotting was used to examine SOX11, ZNF282, and NGN2 expression. Tubulin was used as internal control. **E**,**F** SOX11 and NGN2 were transiently transfected in miR-211-overexpressing cells. Western blotting was used to reveal SOX11 and NGN2 expression after transfection. **G** Overexpression of SOX11 or NGN2 in miR-211-overexpressing cells was examined by in vitro artificial BBB to evaluate their BBB adherence ability. Cell number was counted by luciferase activity (3.2 × 10^4^ cells in SOX11 restoration group, 2.0 × 10^4^ cells in NGN2 restoration group, and 7.2 × 10^4^ cells in miR-211-overexpressing group; *P* = 0.0014 and *P* = 0.0006, respectively). **H** Overexpression of SOX11 or NGN2 in miR-211-overexpressing cells was evaluated by seeding the cells on the in vitro artificial BBB to examine their ability of trans-BBB migration. Cell number was counted by luciferase activity (0.2 × 10^3^ cells in SOX11 restoration group, 0.1 × 10^3^ cells in NGN2 restoration group, 3.9 × 10^3^ cells in miR-211 overexpressing group; *P* = 0.0002 and *P* = 0.0002, respectively). **I** Sphere-forming ability of miR-211-overexpressing cells was evaluated through sphere-formation assay after transfection of SOX11 and NGN2. SOX11 and NGN2 restored in miR-211-overexpressing cells were IC injected into NOD-SCID mice. **J** Brain metastatic ability was monitored once a week using IVIS analysis. **K** The overall survival was analyzed using Kaplan–Meier analysis (*P < 0.05; **P < 0.01; ***P < 0.001; NS no significant difference).

The downstream targeting molecules of miR-211 were then investigated through bioinformatic and gene expression microarray analysis. The selection criteria included (1) binding affinity (delta G, lower than −25 KJ/mol) and (2) binding sites of 3′-UTR of targeting molecules (>2). In total, 74 potential targeting molecules were identified (Supplementary Fig. S6C, D). Figure 6A shows that the expression of *SOX11*, *DTX4*, *ZNF282*, and *NGN2* decreased both in miR-211-overexpressing and BrM3 cells compared with control cells. The qRT-PCR indicated that *SOX11*, *DTX4*, and *NGN2* were significantly decreased. *ZNF282* was then excluded from the potential molecules (Fig. 6B). Western blotting showed that SOX11 and NGN2 were significantly decreased. DTX4 was then excluded from the potential molecules (Fig. 6C and Supplementary Fig. S6F). SOX11 and NGN2 were used for further investigation in the current study. Figure 6D and Supplementary Fig. S6E indicate that SOX11 expression decreased 0.3-fold and 0.7-fold in miR-211-overexpressing MDA-MB-231 and HCC1806 cells, respectively, and NGN2 expression decreased 0.4- and 0.8-fold in miR-211-overexpressing MDA-MB-231 and HCC1806 cells, respectively, compared with controls. In addition, SOX11 and NGN2 expression in BrM3 cells increased 4.2- and 2.1-fold, respectively, in miR-211-knockdown cells compared with controls. To further investigate the specific regulation of miR-211 on SOX11 and NGN2, reporter assay was conducted. Reporter assay indicated that cells treated with miR-211 mimics significantly decreased the luciferase activity compared with NC mimics-treated groups (Supplementary Fig. S7). These results suggested that miR-211 can specifically regulate SOX11 and NGN2 expression. We concluded that SOX11 and NGN2 were the downstream targets of miR-211 in TNBC cells.

**Fig. 7:**
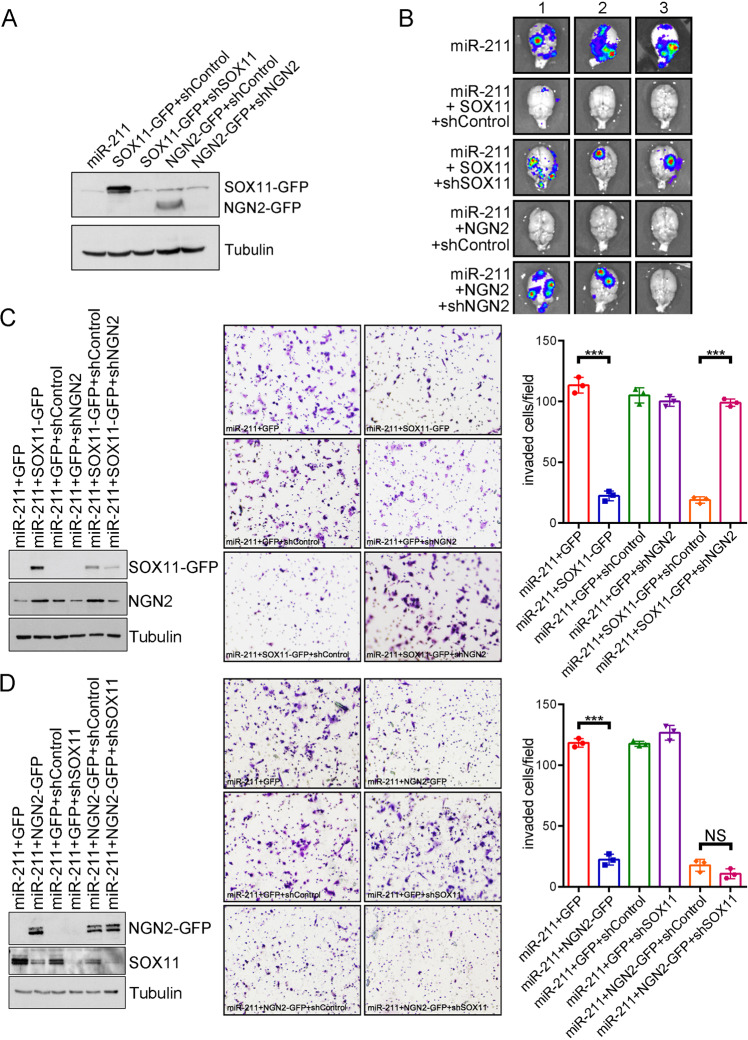
The miR-211-SOX11-NGN2 pathway mediates miR-211-dependent cell invasion in vitro. **A** The protein levels of SOX11-GFP and NGN2-GFP was investigated by western blotting in miR-211 overexpressing MDA-MB-231 cells transiently transfected with (i) parental; (ii) SOX11-GFP and shControl; (iii) SOX11-GFP and shSOX11; (iv) NGN2-GFP and shControl; (v) NGN2-GFP and shNGN2 plasmids. **B** Four weeks after IC injection, the brains were isolated and detected the exist of metastasis cells by IVIS ex vivo. **C** Western blotting indicated the relative expression of SOX11 and NGN2 in different groups. Trans-well analysis was used to evaluate the invasion abilities of miR-211 overexpressing MDA-MB-231 cells transiently transfected with (i) GFP; (ii) SOX11-GFP; (iii) GFP + shControl; (iv); GFP + shNGN2; (v) SOX11-GFP + shControl; (vi) SOX11-GFP + shNGN2 48 h after transfection. **D** Western blotting indicated the relative expression of SOX11 and NGN2 in different groups. Trans-well analysis was used to evaluate the invasion abilities of miR-211 overexpressing MDA-MB-231 cells transiently transfected with (i) GFP; (ii) NGN2-GFP; (iii) GFP + shControl; (iv) GFP + shSOX11; (v) NGN2-GFP + shControl; (vi) NGN2-GFP + shSOX11 48 h after transfection (*** *P* < 0.001; NS no significant difference).

To further investigate whether miR-211 mediated brain metastasis in vitro and in vivo through the SOX11 and NGN2-dependent pathway, restoration experiments were performed. Plasmids with SOX11 and NGN2, which lacked 3′-UTRs, were transiently transfected into miR-211-overexpressing cells and BrM3 cells. Figure 6E, F and Supplementary Fig. S8A show that SOX11 and NGN2 levels were significantly increased in both cell lines. The BBB adherence and trans-BBB migration abilities were significantly attenuated after SOX11 and NGN2 restoration in miR-211-overexpressing and BrM3 groups, respectively (Fig. 6G, H and Supplementary Fig. S8B, C). According to these results, miR-211-mediated BBB adherence and trans-BBB migration abilities were dependent on SOX11 and NGN2. Sphere numbers were also significantly decreased in SOX11 and NGN2 restoration groups (Fig. 6I, sphere number: 30 ± 1.3 in the SOX11 restoration group; *P* = 0.0004, 12 ± 0.8 in the NGN2 restoration group; *P* = 0.0002, 59 ± 1.4 in the miR-211-overexpressed group). Figure 6J shows that brain colonization was first detected at 3 weeks after IC injection in the miR-211-overexpressing group, whereas it could not be detected until 5 and 6 weeks after IC injection in SOX11 and NGN2 restoration groups, respectively. Similar results were observed after SOX11/NGN2 restoration in the BrM3 group (Supplementary Fig. S8D). Most importantly, as observed in Fig. 6K, a poor survival rate was observed in the miR-211-overexpressing group compared with SOX11 and NGN2 restoration groups. Sixty days after IC injection, 25% of mice from the miR-211 overexpression group survived, whereas 100% of mice from both SOX11 and NGN2 restoration groups survived (*P* = 0.03). Similar results were observed after SOX11/NGN2 restoration in the BrM3 group (Supplementary Fig. S8E, *P* = 0.0014). To further investigate the specific role of SOX11 and NGN2 in miR-211 overexpressing mediated brain metastasis in vivo. The shSOX11 and shNGN2 were conducted to downregulate SOX11 and NGN2 expression in SOX11 and NGN2 restoration groups, respectively. The expression of SOX11 and NGN2 was decreased after shRNAs treatment (Fig. 7A). The brain metastasis was observed within 4 weeks compared with SOX11 and NGN2 restoration groups after the SOX11 and NGN2 downregulation (Fig. 7B). High and low SOX11/NGN2-expressing miR-211-overexpressing cells were intracardiac injected into mice. Five weeks after IC injection, all mice injected with miR-211 overexpression cells had brain metastasis. The brain metastasis rate of mice of high SOX11 expression miR-211 group was 40%, which was less than the low SOX11 expression miR-211 group, 60%. The brain metastasis rate of mice of high NGN2 expression miR-211 group was 0%, which was less than the low NGN2 expression miR-211 group, 67%. According to the results, the brain metastasis suppression ability of SOX11 and NGN2 was in a dose-dependent manner (Supplementary Fig. S8F, G). To investigate the logical connection between SOX11 and NGN2 in miR-211 mediated invasion. Western blotting indicated the relative expression of SOX11 and NGN2 in different groups (Fig. 7C, D). The results showed that overexpression of SOX11 and NGN2 significantly decreased miR-211 mediating cell invasion. The SOX11 suppressed cell invasion ability was restored when downregulated the NGN2 expression at the same time. However, the NGN2 suppressed cell invasion ability was not restored when downregulated the SOX11 expression at the same time (Fig. 7C, D). These results suggested that NGN2 is the downstream molecule of SOX11. Taken together, our results suggested that miR-211-mediated brain metastasis and subsequent death may occur through the miR-211-SOX11- NGN2-dependent axis.

## Discussion

**Fig. 8:**
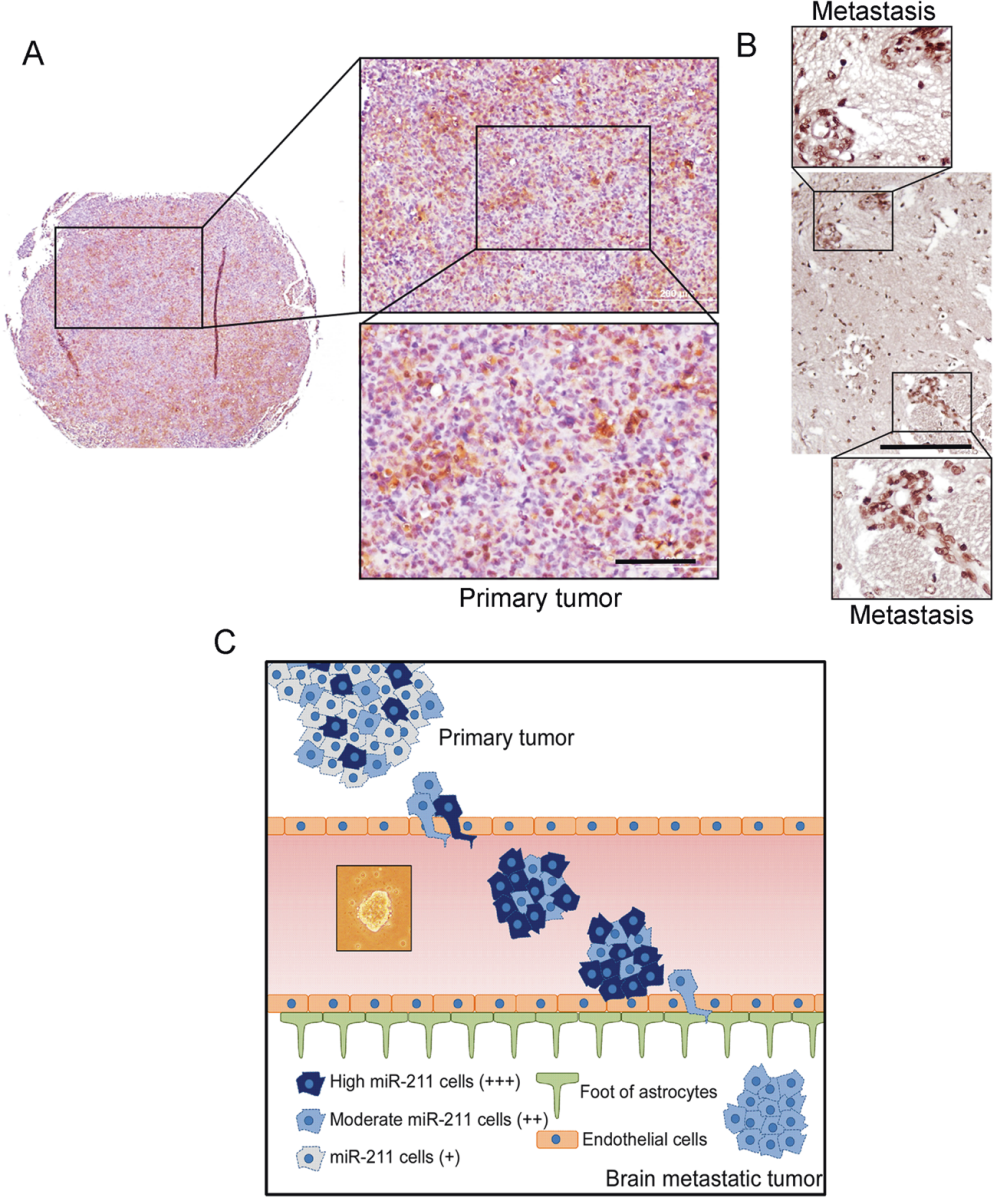
A subpopulation of primary tumor cells with high miR-211 expression mediates brain metastasis in TNBC. **A,** **B** MiR-211 expression was detected by ISH in MDA-MB-231 orthotopically injected xenografted tissues. Metastatic lesions were detected by the IVIS system ex vivo to confirm the tumor lesions. Brown color indicated that the miR-211 signals were eventually visualized by 3,3-diaminobenzidine (DAB) staining, followed by counterstaining with hematoxylin indicting nuclear staining, **C** The diagram shows the dynamic brain metastatic process of triple-negative breast cancer cells with different levels of miR-211. Cells with high/moderate miR-211 levels were adhered to blood vessels and collectively migrated to the blood–brain barrier (BBB). Cells with high miR-211 levels adhered to the endothelial cells of the BBB. The tumor cells further formed distant metastases in the brain. Scale bar = 100 μm (**A**); 200 μm (**B**).

In this study, the isolated brain-tropic cells cause early brain metastasis and poor survival in mice. Identification and functional characterization of high miR-211 promoted early brain metastasis and caused poor survival in vivo through regulating adherence to the BBB, trans-BBB abilities, and stemness properties. Our results provided evidence to show that high miR-211 promotes brain metastasis at different metastatic stages. At the primary site, MDA-MB-231 and BrM3 cells were orthotopically injected. The primary tumors were collected to performed qRT-PCR (Fig. 2D) and ISH (Fig. 4A). High miR-211 was detected in metastatic tissues. We also observed ISH to show that different expression levels of miR-211 can be detected in the primary sites, which indicates that a subpopulation of high miR-211 may be able to metastasize to the brain (Fig. 8A). The high miR-211 cells contain stemness properties (Fig. 5B–H), which is critical for the tumor cell survival in the circulation. High plasma miR-211 expression was correlated with the brain metastasis in our xenograft mice model (Fig. 3B). At the metastatic sites, high miR-211 enhanced the cell adhesion to the endothelial cells of BBB and promoted trans-BBB migration ability (Fig. 4H–J). High miR-211 was expressed in the metastatic regions (Figs. 4A and 8B). Together with the above evidence, cells with high and moderate miR-211 harbor high intravasation ability and collective cells rolling together in blood circulation. Once the collective tumor cells attach to the endothelium of the BBB, the high miR-211 cells anchor and penetrate through the BBB, and form brain metastatic tumors (Fig. 8C).

The functional role of miR-211 in tumorigenesis remains controversial. As an oncomiR, miR-211 suppresses the expression of *RRM2* to enhance metastasis and recurrence in patients with colorectal cancer who harbor a *K-RAS* gene mutation [27]. miR-211 enhances the oncogenesis of carcinogen-induced oral carcinoma by increasing antioxidant activity and downregulating TCF12 [28]. As a tumor suppressor miRNA, miR-211 has been reported to suppress tumor cell proliferation, invasion, and migration in vitro and in vivo in BCs [29]. In our study, the high expression of miR-211 promoted brain-tropic metastasis and caused poor survival in vivo. Knockdown of miR-211 in BrM cells abolished brain metastasis and prolonged survival in vivo (Fig. 2).

A previous study demonstrated that the overexpression of miR-141 injected into the tail vein promotes the brain metastasis of BC [24]. In our study, through miRNA array analysis, the miR-141 level significantly increased in brain-tropic cells (BrM-831) compared with MDA-MB-231 parental cells. Our results also showed that the overexpression of miR-141 promoted brain-tropic metastasis through IC injection; however, Kaplan–Meier analysis showed that miR-141 did not lead to poor survival in vivo. Therefore, miR-211, and not miR-141, was selected for further investigation in our study.

In our study, two transcriptional factors, SOX11 and NGN2, were identified downstream of miR-211 to promote the miR-211-dependent brain-tropic metastasis of TNBC (Fig. 6). Previous studies showed that SOX11 is significantly increased in tamoxifen-resistant MCF7 cells. Tamoxifen-resistant cells promote migration and invasion via Slug-dependent EMT. High SOX11 is correlated with poor prognosis in BC patients [30]. Bioinformatics and clinical association investigation indicates that SOX11 promotes migration and invasion in basal-like BC [31]. Nuclear SOX11 is correlated with lymph node metastasis in BC [32]. A previous study also indicates that SOX11 plays tumor-suppressive roles in cancer development. Overexpression of SOX11 delays cancer progression by repressing the development of cancer-initiating cells and reducing cancer metastasis [33]. SOX11 prevents tumorigenesis of glioma-initiating cells [34]. The functional roles of SOX11 and NGN2 in cancers remain controversial. According to our results, SOX11 and NGN2 serve as inhibitors of miR-211-induced brain metastasis; however, the downstream molecules regulated by SOX11 and NGN2 remain unclear, and further investigation to expose their mechanisms is warranted.

Circulating miRNAs used as prognostic markers have been observed in many cancers, including BC. In our study, results indicated that when miR-211 expression exceeds the cut-off value, brain metastasis may be detectable (Fig. 3B, C). Therefore, detection of plasma miR-211 level may help physicians to identify TNBC patients with brain metastasis. A personalized therapeutic strategy may be able to prevent brain metastasis of TNBC for better treatment outcomes in the future.

## Materials and methods

### Cell lines

The human BC cells, MDA-MB-231, HCC1806, and different organ-tropic cells, including of LM2-4175 (lung metastasis), and BrM-831 (brain metastasis) were used in this study. The BrM-831 cells are isolated by two rounds in vivo selection, and the cells show a significant increase in brain metastasis activity, but no increase in bone or lung metastatic activity compared to the parental population [14, 35]. The LM2-4175 cells show a significant increase in lung metastasis activity [36]; MDA-MB-231 and different organ-tropic cells, including of LM2-4175 (lung metastasis), and BrM-831 (brain metastasis) were cultured in the Dulbecco’s Modified Eagle Medium supplemented with 10% cosmic calf serum (HyClone, Logan, UT, USA), and 1% penicillin–streptomycin (P/S) (Caisson Labs, Smithfield, UT, USA). HCC1806 was cultured in the RPMI 1640 Medium supplemented with 10% cosmic calf serum, and 1% P/S. All cells were maintained at 37 °C in a humidified atmosphere of 5% CO_2_.

### Statistical analyses

All observations were confirmed by at least three independent experiments. Data are expressed as mean ± SD. The association between OS and DFS was analyzed using log-rank Kaplan–Meier analysis with SPSS software. The results were compared using an unpaired two-tailed Student’s *t*-test and multiple *t* test. All tests were two sided, and a *P* value < 0.05 was considered statistically significant. The *χ*^2^ test was used to calculate the significance of clinical specimens of patients with and without brain metastasis. The ROC curve was used to examine the prognostic power, which was represented by the AUC. Data were analyzed using GraphPad Prism 6 software (GraphPad Software, San Diego, CA, USA).

More materials and methods are included in the Supplementary files due to the space limit (Supplementary Materials and Methods).

## Supplementary information

Supplementary information

Figure S1

Figure S2

Figure S3

Figure S4

Figure S5

Figure S6

Figure S7

Figure S8
