# Supplementary material for: MiR-211 determines brain metastasis specificity through SOX11/NGN2 axis in triple-negative breast cancer
Source: Oncogene. 2021 Feb 3;40(9):1737–51. doi: 10.1038/s41388-021-01654-3 (PMC7932919; doi:10.1038/s41388-021-01654-3)
Supplement: Supplementary file 1 — Supplementary information [file 41388_2021_1654_MOESM1_ESM.docx]

### Supplementary Materials & Methods

**In vivo *selection***

All animal experiment protocols were approved by the Institutional Animal Care and Use Committee in National Cheng Kung University (IACUC NO.:106098, 107198). Three-dimensional ultrasound was used to locate the left ventricle of the heart of NOD/SCID female mice. MDA-MB-231 cells, which carry luciferase, were IC injected into the hearts of the mice. IVIS was performed to monitor the distribution of tumor cells in circulation after injection. Metastatic tumor cells were monitored using IVIS once a week. After the formation of metastatic brain tumors, the metastatic cells were isolated from brains and referred to as BrM1 cells. This process was repeated twice to acquire BrM2 and BrM3 cells.

***Xenograft animal model***

The brain metastasis mice model of NOD/SCID female age-matched mice aged 4–6 weeks were obtained from Laboratory Animal Center (National Cheng Kung University) and randomly used for xenograft studies. 1 × 10^5^ BCCs/ 100μl HBSS were injected into the left ventricle of the heart of the mice. Tumor metastasis and growth were detected using the IVIS system once a week. For blinding analysis of the results, the IVIS images were collected by the technicians of the Laboratory Animal Center. The animals were sacrificed at 7–10 weeks, depending on their condition. The organs were then collected in a 10-cm dish for IVIS *ex vivo* detection. The brain metastatic capacity of parental MDA-MB-231 cells and its derived cells including *in vivo* isolated cells (BrM1, BrM2, and BrM3), and *in vitro* isolated cells (BBB adherent cells and trans-BBB cells) was evaluated by IC injection.

For establishing an orthotopic breast cancer model, 1 × 10^5^ BCCs/ 50μl HBSS were injected into the mammary fat pads of NOD/SCID female mice. Tumor growth was detected using IVIS once a week. In approximately 5 weeks, the primary tumor was surgically removed, and the incision was closed. The animals were sacrificed at 15–20 weeks. The organs were then collected and subjected to IVIS detection *ex vivo*.

The plasma of mice was collected by submandibular blood collection once a week. The serum was separated from the blood by centrifugation at 3000 rpm for 30 min and stored at −80°C.

***Clinical specimens***

Human plasma specimens were obtained from Kaohsiung Veterans General Hospital (Kaohsiung, Taiwan), and National Cheng Kung University Hospital (Tainan, Taiwan). All patients provided written informed consent, and the protocol was approved by the Institutional Review Boards of all the hospitals (VGHKS12-CT9-07, B-ER-105-308). A plasma sample was collected from each patient during treatment. The 130 plasma samples were stored in liquid nitrogen until use. The patients were subdivided into TNBC (ER-/PR-/ HER2-) and non-TNBC groups according to the different tumor grades and metastasis statuses.

Human BC tissue microarrays (TMA-BCs), mainly from samples from two clinical cohorts of patients, were collected and prepared from Kaohsiung Veterans General Hospital archives, and primary breast cancer tissues were collected with patients’ informed consent and approval by Institutional Reviewed Board (VGHKS13-CT11-18, VGHKS14-CT7-15). The molecular subtypes of breast cancer patients’ specimen were evaluated by IHC while enrolled to establish a tissue microarray. The outcomes of molecular subtypes were confirmed by the pathologists (**Fig. S4A** and **Table S1**). All tissue sections with tumor parts were fixed by 10% formalin, dehydrated, embedded in paraffin, and further histologically examined for the presence of tumor by ISH.

***Extraction and detection of circulating miRNAs***

Circulating miRNAs were extracted using the Direct-zol™ RNA MicroPrep Kit (Zymo Research, Irvine, CA, USA), and 10–50 ng/µL of total RNAs were reverse transcribed into cDNA based on poly A-tail directed reverse transcription, as previously described. Expression was analyzed by qPCR using the SYBR Green master mix (KAPA), and U6 was used as the internal control.

***Stable cell-line generation***

The full-length pre-miRNA-211 and pre-miRNA-141 were constructed into the pCDH-CMV-mcs-EF1α-RFP-puro vector (System Biosciences, Palo Alto, CA, USA). Next, lipofectamine 2000 was used to transfect the plasmids into the MDA-MB-231 to establish miRNA-211- or miRNA-141-overexpressing cells. Subsequently, stable clones were isolated through puromycin selection at 48-h after transfection and enriched through fluorescence-activated cell sorting of the RFP++ cells. Fluorescence intensities were analyzed using Cell Quest Software (BD Biosciences).

***Western blotting***

The cells were homogenized in 2× sample buffer, and the lysates were heated at 95°C for 10 min, placed on ice for a few minutes, and then stored at −80°C. The protein expression levels of SOX11 (GeneTex, GTX129501), NGN2 (GeneTex, GTX129258), ZNF282 (GeneTex, GTX118812), α-tubulin (Santa Cruz, sc-3948), Oct4 (Cell Signaling, 2750), Nestin (Cell Signaling, 33475), CD44 (R&D, #BBA10), TCF4 (GeneTex, GTX61704), fibronectin (Abcam, ab6328), beta-catenin (GeneTex, GTX101435), Twist (GeneTex, GTX127310), SLUG (GeneTex, GTX128796), SNAIL (Abcam, ab53519), VIMENTIN (BD, 550513), ABCB1 (Cell Signaling, 13342), and ABCG2 (Genetex, GTX100437) were measured using immunoblotting analysis. The intensities of blots were quantified using ImageJ.

***RNA extraction, cDNA synthesis, and qRT-PCR***

### Total RNAs were collected from human BCCs by the TRIsure^TM^/chloroform method and were stored at −80°C. cDNA was synthesized from 1 µL of total RNA by using the M-MLV reverse transcriptase system kit (Invitrogen, Carlsbad, CA, USA), according to the manufacturer’s instructions. The mRNA expression levels of target genes were measured using Fast SYBR Green Master Mix (Kapa Biosystems, Wilmington, MA, USA) and the StepOne real-time PCR system (Applied Biosystems)*.* All reactions were performed in triplicate, and relative gene expression was calculated by the 2^−ΔΔ^CT method. *GAPDH* was used as the internal control.

***Soft agar assay***

Soft agar was prepared in the six well plates. The cells were seeded at the density of 1 × 10^4^ per well to 2 mL DMEM culture medium with 0.3% agarose gel, and split onto DMEM culture medium with 0.5% low-melting agarose gel. The plate was incubated at 37℃ for 14 days to allow colonies formation, and count the cell colonies with bright field microscopy.

***Cell viability***

Cell growth assay were performed by seeding 1 × 10^3^ cells/well in the 96 well plates with DMEM medium containing 10% CCS. Time points were taken at 0, 24, 48, 72 hours and cells were washing with HBSS. Then, cells/well were added 100 uL 20% 3-(4,5-Dimethylthiazol-2-yl)-2,5-diphenyltetrazolium bromide (MTT) reagent diluted with culture medium and incubated at 37℃ for 2 hours. Remove the 20% MTT solution from 96 plate and add 50 uL DMSO solution at 37℃ for 2 minutes. Solution from the bottom chamber was collected, and absorbance was measured at 570 nm by Elisa reader.

***MicroRNA array***

MDA-MB-231, LM2-4175, and BrM-831 were subjected to array analysis. TaqMan Array MicroRNA Cards were used to analyze the unique miRNA profiles, and the experiment was conducted by Topgen Biotechnology Co., Ltd.

***Gene expression microarray analysis***

MDA-MB-231 cells, miR-211-overexpressing cells, and BrM3 cells were collected and lysed by TRIsure^TM^. Gene expression array (SurePrint G3 Human Gene Expression 8x60K Microarray) was performed by Welgene Biotech Co., Ltd; 0.2 μg of total RNA was amplified using a Low Input QuickAmp Labeling Kit (Agilent Technologies, USA) and labeled with Cy3 (CyDye, Agilent Technologies, USA) during the *in vitro* transcription process. A total of 0.6 μg of Cy3-labled cRNA was fragmented to an average size of approximately 50–100 nucleotides by incubation with fragmentation buffer at 60°C for 30 min. Correspondingly, fragmented labeled cRNA was then pooled and hybridized to the Agilent SurePrint Microarray (Agilent Technologies, USA) at 65°C for 17 h. After washing and drying by nitrogen gun blowing, the microarrays were scanned using an Agilent microarray scanner (Agilent Technologies, USA) at 535 nm for Cy3. Raw signal data were normalized by quantile normalization for identifying differentially expressed genes. All datasets will be publicly available for querying and downloading through the GEO database when this paper is accepted.

***cDNA synthesis and qRT-PCR for miRNA***

In total, 100ng of total RNA was mixed with poly (A) polymerase (Takara), 5X polyA buffer, and MnCl_2_ (25mM) in a sterile 0.2-mL PCR tube, and RNase-free water was added to make a final volume of 20 µL. The mixture was then incubated at 37°C for 30 min for the polyA reaction. The RTQ primer (5’- CGAATTCTAGAGCTCGAGGCAGGCGACATGGCTGGCTAGTTAAGCTTGGTACCGAGCTCGGATCCACTAGTAGTCCTTTTTTTTTTTTTTTTTTTTTTTTTVN -3’) (1 µM), design based from previously described [1], and 1 µL of poly A product was mixed, and the mixture was heated at 65°C for 5 min, followed by quick chilling on ice. Next, 1 µL of 10mM dNTP, 4 µL of 5X first-strand buffer, 2 µL of 0.1M DTT, and 0.5 µL (200units) of M-MLV reverse transcriptase were added. The contents of the tube were mixed gently and incubated at 37°C for 2 min; next, they were incubated at 25°C for 10 min and 37°C for 50 min, and the reaction was inactivated by heating the mixture at 70°C for 15 min in a PCR Thermal Cycler (BIO-RAD). cDNA was stored at −20°C until use. The expression levels of miRNAs were measured using the Fast SYBR Green Master Mix and a StepOne real-time PCR system*.* The results were normalized to U6.

***Sphere-formation assay and Sphere intracardiac injection***

In total, 1 × 10^3^ to 5 × 10^3^ cells/100 µL were cultured in a 96-well ultra-low adhesion culture dish containing DMEM/F-12 (Gibco) with 20 ng/mL of rhEGF (PeproTech), 10 ng/mL of rhbFGF (PeproTech), N2 supplement (Gibco, 17502-048), and 1% P/S at 37°C for 10–14 days. Sphere number was counted using a microscope (Olympus IX71) and ImageJ to calculate the efficiency of sphere formation and sphere numbers. For sphere intracardiac injection, one thousand spheres were collected, without dissociation, and then performed IC injection. Each sphere contained about 8-12 cells. IVIS system was used to monitor the brain metastasis weekly.

**In vitro *BBB mimicry system***

HUVECs were co-cultured with human primary astrocytes (ScienCell) on poly-L-lysine- and gelatin-coated 24-well culture plates for 3 days to establish BBB mimicry. Cancer cells were seeded at a density of 5 × 10^5^ cells/well on the BBB mimicry system at 37°C for 5 min. After 5 min, the cell suspensions were removed from the wells and washed with 1× HBSS thrice. Subsequently, cells attached to the 24 wells were trypsinized by 1× trypsin incubated at 37°C for 5 min. Cell suspensions were collected in 15-mL centrifuge tubes, and cells were lysed using the Dual-Luciferase Reporter Assay System (Promega); luciferase activity represented as the cell numbers and was detected using an ELISA plate reader.

***Trans-BBB migration assays***

Transwell culture inserts with 8-µm pores (BD Biosciences) were coated with poly-L-lysine (1 mg/mL) overnight at 37°C, washed four times with distilled water, and coated with 0.2% gelatin (Sigma) for 30 min. The coated inserts were placed upside-down in a 12-well plate (GeneDirex), and 10^5^ primary human astrocytes (ScienCell) were plated on the membrane surface. Astrocytes were fed with culture medium every 15 min for 5 hr, and the inserts were then flipped and placed in 24-well plates. In total, 50,000 endothelial cells were plated on the upper chamber of the inserts, and the cultures were placed in the incubator. Three days later, the permeability of the barriers was tested using sodium fluorescein dye (0.01%) diluted with the culture medium. The dye (0.01%) was added to the upper chamber and the culture was incubated for 15 and 30 min at 37°C. The medium in the bottom chamber was collected, and absorbance was measured using the ELISA plate reader.

Cancer cells (2 × 10^5^ cells/100 µL) were seeded in the top chamber; they were coated with human astrocytes and HUVECs for 3 days and cultured at 37°C for 48 h. After 48h, trans-BBB cells on the lower side of the filter membrane were trypsinized by 1× trypsin incubated at 37°C for 5 min. Cells were collected in 15-mL centrifuge tubes and lysed using Dual-Luciferase Reporter Assay system (Promega); luciferase activity represented as the cell numbers and was detected using an ELISA plate reader.

**In situ *hybridization (ISH)***

IsHyb In Situ Hybridization Kit (BioChain, Newark, CA, USA) was used according to the manufacturer’s instructions. Briefly, paraffin-embedded xenograft tissues and human TMAs were deparaffinized and rehydrated, followed by 4% paraformaldehyde fixation in 1× DEPC-PBS. The slides were treated with 10 μg/mL of proteinase K (BioChain) and incubated with a pre-hybridization solution at 50°C for 4 h before hybridization with Custom LNA™ has-miR-211-5p or U6 probe (Exiqon, Vedbaek, Denmark) at 55°C overnight. Two visualized methods were used to detecte the signals, (1) the slides were then washed with saline sodium citrate buffer, followed by incubation with alkaline phosphatase-conjugated anti-digoxigenin antibody at room temperature for 4 h. The signals were eventually visualized by nitro blue tetrazolium/5-bromo-4-chloro-3-indolyl-phosphate (NBT/BCIP) staining, followed by counterstaining with Nuclear Fast Red. (2) The signals were eventually visualized by 3,3′-diaminobenzidine (DAB) staining (DAB, Sigma), followed by counterstaining with hematoxylin.

***Invasion ability analysis***

The trans-well was first coated with Matrigel (1mg/mL, BD Bioscience). Coated trans-well placed in the 24 well plates and each well contained with 1mL of culture medium containing 10% CCS. Each top of chamber was filled with 100 uL cell suspensions (1 × 10^5^ cells/well) and incubated at 37℃ for 12 hours. Lower side of the membrane was fixed with methanol and staining by 10% Giemsa reagent (Sigma-Aldrich, St. Louis, MO).

***Reporter assay***

The SOX11 3’UTR and NGN2 3’UTR were cloned into the downstream of luciferase gene in pMIR-REPORT luciferase vector (Thermo Fisher Scientific). The 3 × 10^5^ cells/well HEK293T were co-transfected with SOX11 3’UTR or NGN2 3’UTR, miR-211 mimics and Renilla luciferase plasmid as normalize control using Lipofectamine 2000. Reporter assay was performed at 24 hours post transfection using the Dual-Luciferase Reporter Assay system (Promega).

**Supplementary reference**

1 Ro S, Park C, Jin J, Sanders KM, Yan W. A PCR-based method for detection and quantification of small RNAs. *Biochemical and biophysical research communications* 2006; 351: 756-763.

**Supplementary Table**

| **Table S1. The miR-211 expression level in BC patients with different molecular subtypes** | | | |
| --- | --- | --- | --- |
|  |  |  |  |
| **Molecular Subtype of BC** | **Patient number** | **high miR-211** | **low-miR-211** |
| **BC** | 484 | 230 | 254 |
|  |  |  |  |
| **non-TNBC** | 229 | 99 | 130 |
| ER^+^ | 20 | 7 | 13 |
| PR^+^ | 8 | 2 | 6 |
| HER2^+^ | 50 | 24 | 26 |
| ER^+^PR^+^ | 130 | 57 | 73 |
| ER^+^HER2^+^ | 7 | 4 | 3 |
| ER^+^PR^+^HER2^+^ | 14 | 5 | 9 |
|  |  |  |  |
| **TNBC** | 255 | 131 | 124 |
|  |  |  |  |
| **Note: There are no PR+HER2+ in our cohorts** | |  |  |

**Supplementary Figure Legends**

### Supplementary Figure 1. Brain-tropic cells have slower tumor growth ability but increase the anchorage independent growth ability. A. MDA-MB-231 cells were IC injected in NOD/SCID female mice. The metastatic tumor cells were tracked once a week using the IVIS system. After tumor colonization in mice brains, the brain metastatic cells were isolated and indicated as BrM cells. After three independent isolations, the BrM1, BrM2, and BrM3 cells were collected for further investigation; B. Cell viability of MDA-MB-231, BrM1, BrM2 and BrM3 were detected by MTT assay; C. The primary tumors were detected through IVIS system during six weeks after tumor orthotopic injection. The signal of primary tumor in the injected site was quantified through IVIS analysis. D. Soft agar assays was used to investigate the anchorage independent growth ability in BrM3 and MDA-MB-231 cells. Colonies were counted by phase microscopy after 14 days seeding. (**P*<0.05; ***P*<0.01)

### Supplementary Figure 2. Differential expression of microRNAs in different organ-tropic cells. A. MicroRNA array was used to investigate the differential expression profiles in different organ-tropic cells (LM2-4175 and BrM-831). The Venn diagram and heat map suggested up- or down-regulated miRNAs in BrM-831 or LM2-4175 cells compared with MDA-MB-231 cells; B. qRT-PCR was used to evaluate the expression levels of 9 miRNAs in the different organ-tropic cells (MDA-MB-231, parental cells; LM2-4175, lung-tropic cells; BrM-831, brain-tropic cells); C. qRT-PCR was used to evaluate the expression of BrM3 cells treated with NC and anti-miR-211 antagomiR at different time points. (**P*<0.05; ***P*<0.01; ****P*<0.001)

### Supplementary Figure 3. MiR-211 overexpression promotes cell invasion and inhibits tumor growth *in vitro* and *in vivo*. A. 1x10^5^ MDA-MB-231-SCR and MDA-MB-231-miR-211 cells were seeded on 6-well plate. The cell numbers were count for 3 days. B. Invasion ability analysis was conducted in (1) MDA-MB-231-SCR; (2) MDA-MB-231-miR-211 cells; (3) BrM3-NC antagomir; (4) BrM3-miR-211 antagomir. C. 1x10^5^ MDA-MB-231-SCR and MDA-MB-231-miR-211 cells were orthotopically injected in mice mammary gland. IVIS system was used to estimate the tumor growth rate weekly. (****P*<0.001)

### Supplementary Figure 4. High miR-211 significantly correlated with poor survival in BC patients. A. Kapan-Meier Analysis was conducted to evaluate the overall survival (OS) and disease-free survival (DFS) in breast cancer patients with different subtypes; B. Overall survival of the 117 patients with BC was analyzed by Kaplan–Meier analysis after stratification by miR-211 expression level; C. High miR-211 was correlated with poor survival of patients with 1262 BC in TCGA datasets

### Supplementary Figure 5. *In vitro* artificial BBB system was established and measured by sodium fluorescence tracking dye. A. HUVECs were co-cultured with human astrocytes, which were seeded on opposite sides of a transwell insert for 3 days. The sodium fluorescence tracking dye (0.01mg/mL) was used to evaluate BBB permeability. The fluorescence intensity represented the integrity of the BBB; B. The standard curve of cell numbers was calculated by detection of luciferase activity in various triple-negative breast cancer cells (MDA-MB-231-scramble, MDA-MB-231-miR-211, MDA-MB-231-GL, and BrM3 cells); C. (1) HCC1806 (2) miR-211 overexpressing HCC1806 were seeding on the *in vitro* artificial BBB to evaluate the adherence ability. Adherence cell numbers would be counted by RFP intensity of HCC1806. (****P*<0.001)

### Supplementary Figure 6. SOX11 and NGN2 are potential miR-211-targeting molecules identified by bioinformatics analysis. A. qRT-PCR was used to evaluate the level of miR-211 after miR-211 overexpression in HCC1806 cells; B. Sphere-formation assay was used to examine stemness ability of parental and miR-211-overexpressing HCC1806 cells; C. miRwalk 2.0 and RNAhybrid 2.0 were used to predict miR-211-regulated genes. The miR-211 binding site numbers in 3′UTR and binding affinity of the predicted genes were calculated; D. Scheme indicates the gene expression microarray analysis performed in this study; E. Western blotting was used to reveal the expression of SOX11, NGN2, and ZNF282 in parental and miR-211-overexpressing HCC1806 cells. F. The protein levels of DTX4, SOX11 and NGN2 were detected by Western blotting after miR-211 overexpression. (***P*<0.01; ****P*<0.001; NS: no significant difference)

### Supplementary Figure 7. MiR-211 specifically regulates SOX11 and NGN2 transcripts. A. The diagram shows the seed regions of miR-211 in SOX11 and NGN2 3’UTR. B. Luciferase reporter assay was conducted to evaluate the binding activity of miR-211 mimics (High: 240nM; Low:160nM) on SOX11 3′-UTR or NGN2 3′-UTR 24 h post transfection. (****P*<0.001; NS: no significant difference)

### Supplementary Figure 8. SOX11 and NGN2 restoration suppress early brain metastasis and prolong survival time *in vivo.* A. Western blotting was used to evaluate the expression of SOX11 and NGN2 in BrM3 cells after protein restoration; B. BrM3 cells with SOX11 or NGN2 restoration were seeded on the *in vitro* BBB system to examine their ability of BBB adherence. Cell number was counted by luciferase activity.(1.1 × 10^4^ cells in SOX11 restoration group, 0.6 × 10^4^ cells in NGN2 restoration group,1.6 × 10^4^ cells in BrM3 group; *P*=0.015 and *P*=0.0002, respectively); C. BrM3 cells with SOX11 or NGN2 restoration were seeded on the *in vitro* system to examine their ability of trans-BBB migration. Cell number was counted by luciferase activity.(0.8 × 10^2^ cells in SOX11 restoration group, 0.5 × 10^2^ cells in NGN2 restoration group, 2.3 × 10^2^ cells in miR-211-overexpressing group; *P*=0.023 and *P*=0.0003, respectively); D. SOX11- and NGN2-restored BrM3 cells and BrM3 cells were IC injected into NOD/SCID mice. Their brain metastatic ability was monitored once a week using IVIS; E. Kaplan–Meier analysis was used to evaluate the overall survival in NOD-SCID mice IC injected with SOX11- and NGN2-restored BrM3 cells. F. Western blot was used to evaluate the levels of SOX11-GFP and NGN2-GFP in miR-211 overexpressing cells transiently transfected with different dose of SOX11-GFP and NGN2-GFP plasmids. G. High and low expression of SOX11/NGN2 cells were intracardiac injected into mice. IVIS system was used to monitor the brain metastasis weekly. (***P*<0.01; ****P*<0.001)
