# Supplementary figures and images for: MiR-211 determines brain metastasis specificity through SOX11/NGN2 axis in triple-negative breast cancer
Source: Oncogene. 2021 Feb 3;40(9):1737–51. doi: 10.1038/s41388-021-01654-3 (PMC7932919; doi:10.1038/s41388-021-01654-3)

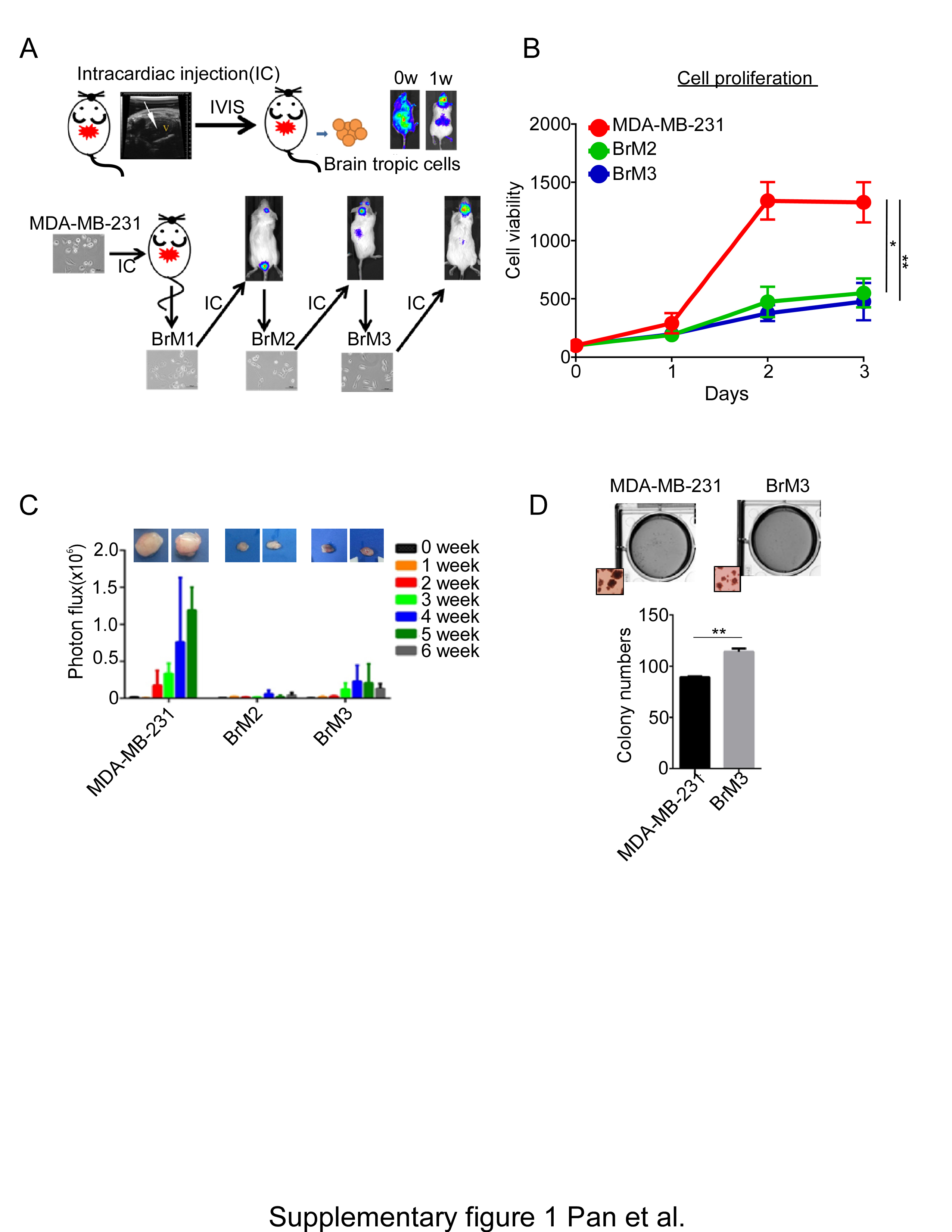

Supplement: Supplementary file 2 — Figure S1 [file 41388_2021_1654_MOESM2_ESM.jpg]

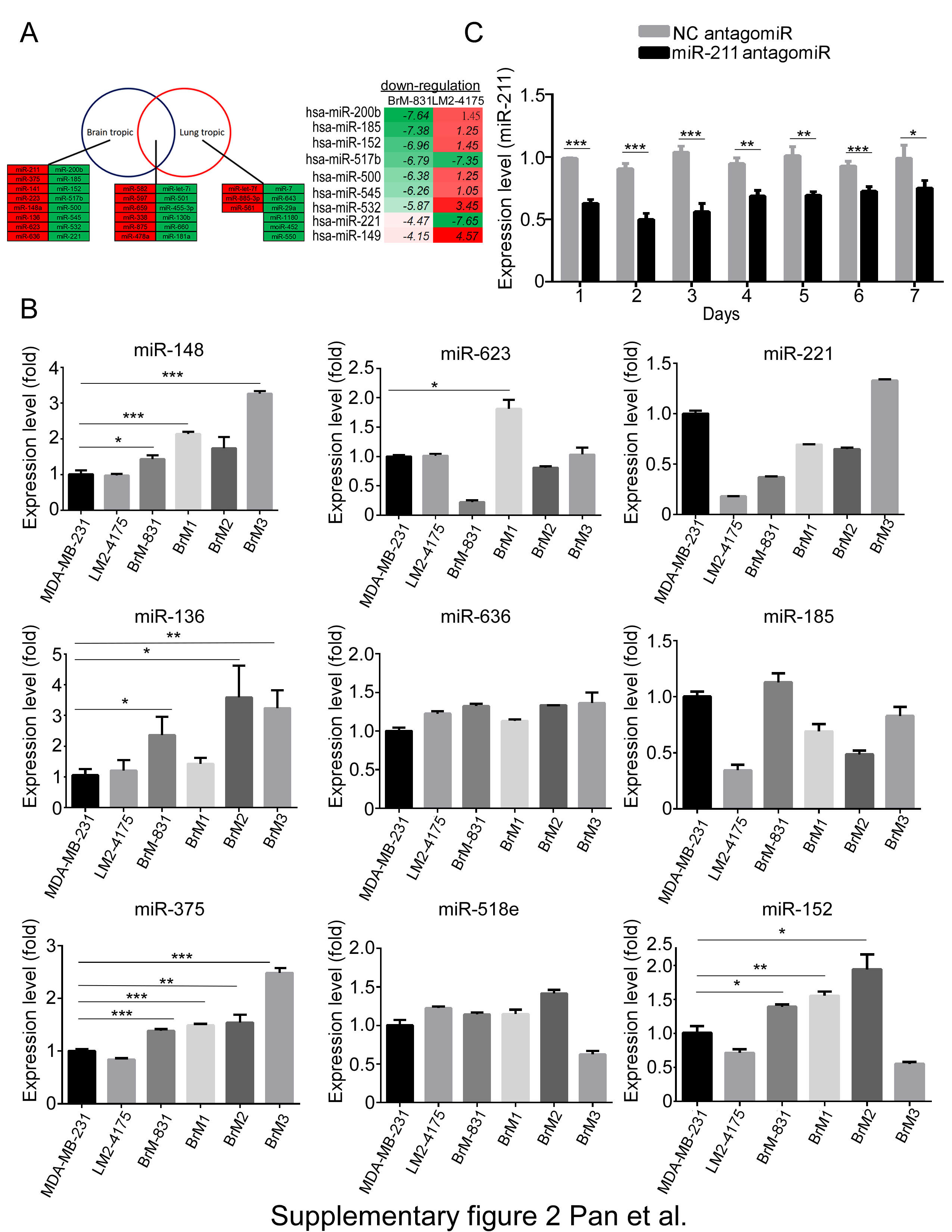

Supplement: Supplementary file 3 — Figure S2 [file 41388_2021_1654_MOESM3_ESM.jpg]

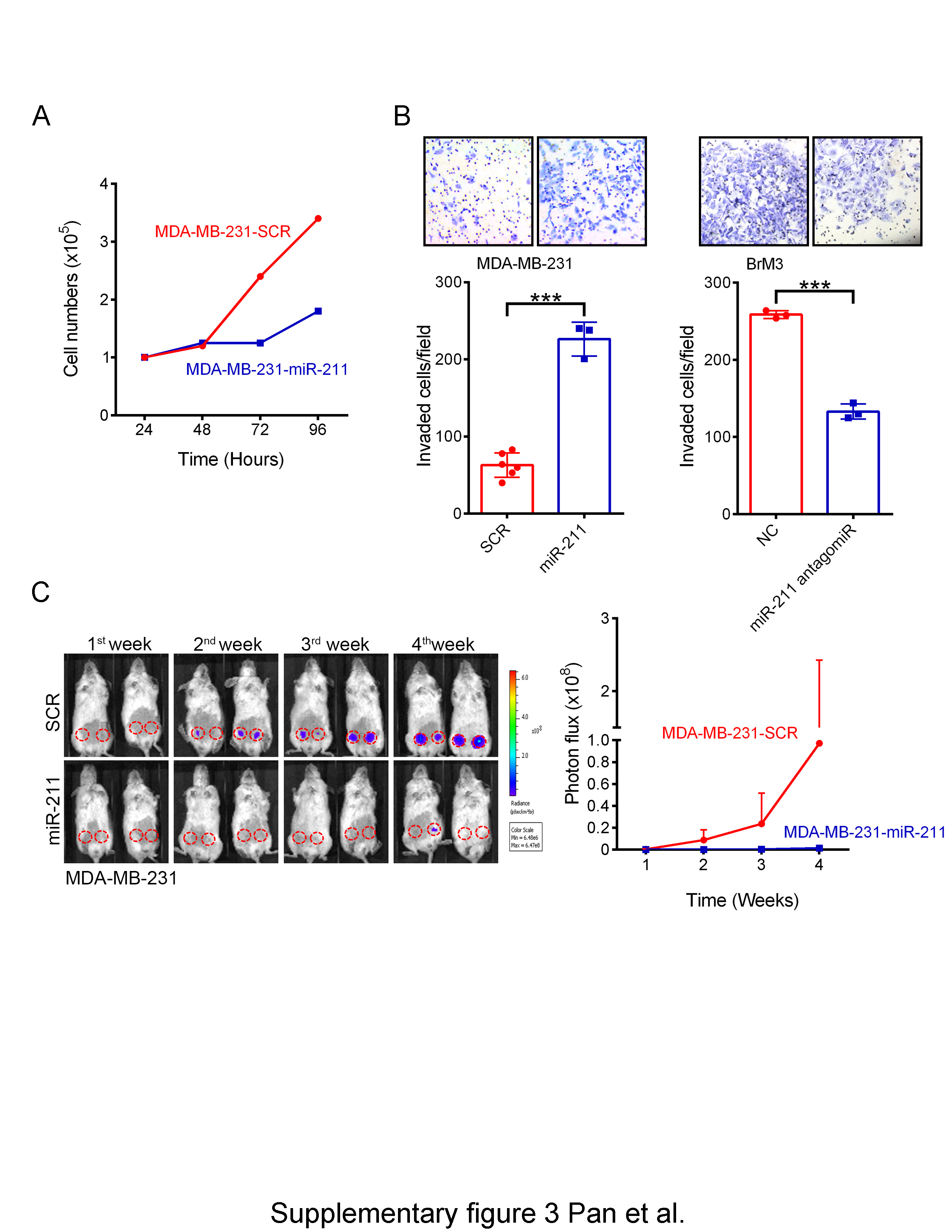

Supplement: Supplementary file 4 — Figure S3 [file 41388_2021_1654_MOESM4_ESM.jpg]

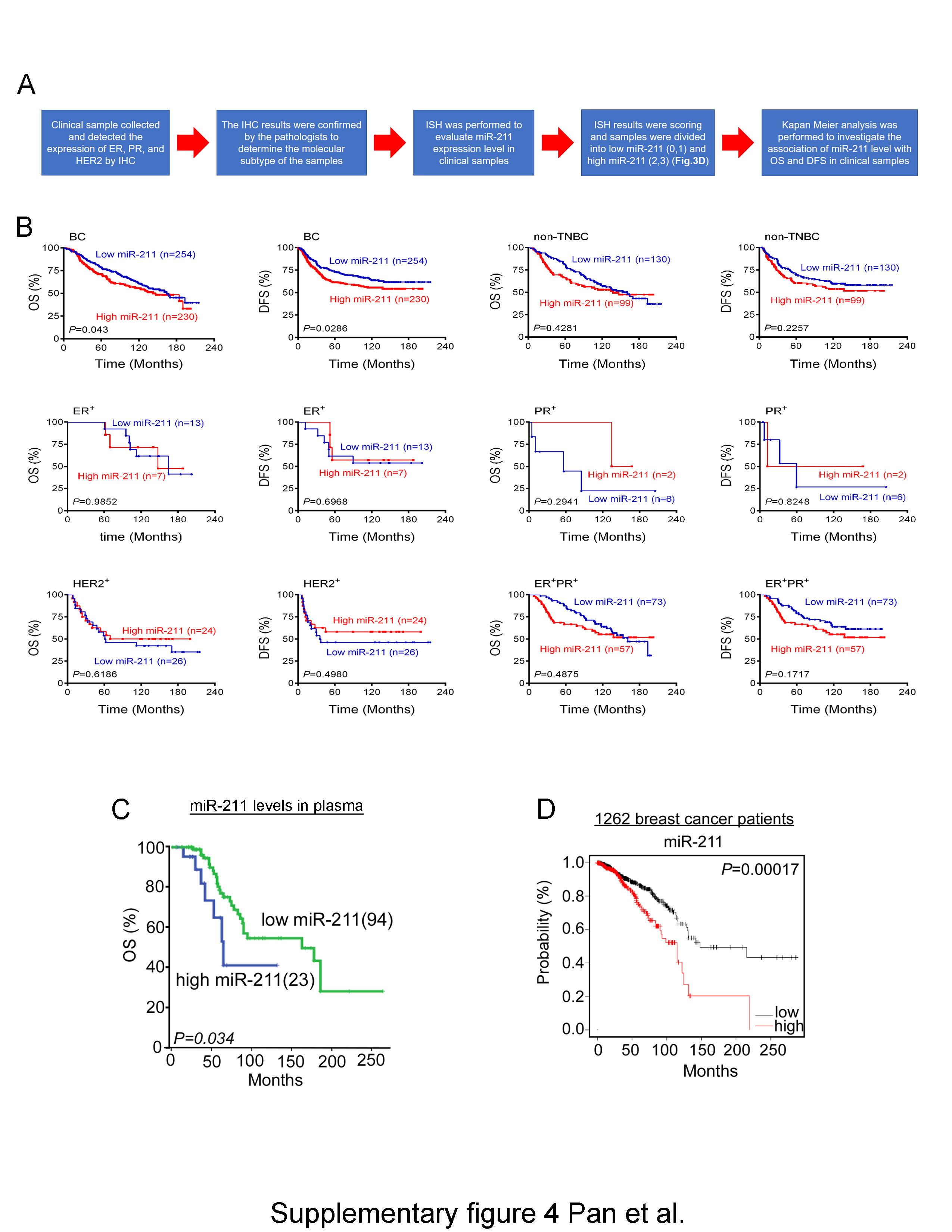

Supplement: Supplementary file 5 — Figure S4 [file 41388_2021_1654_MOESM5_ESM.jpg]

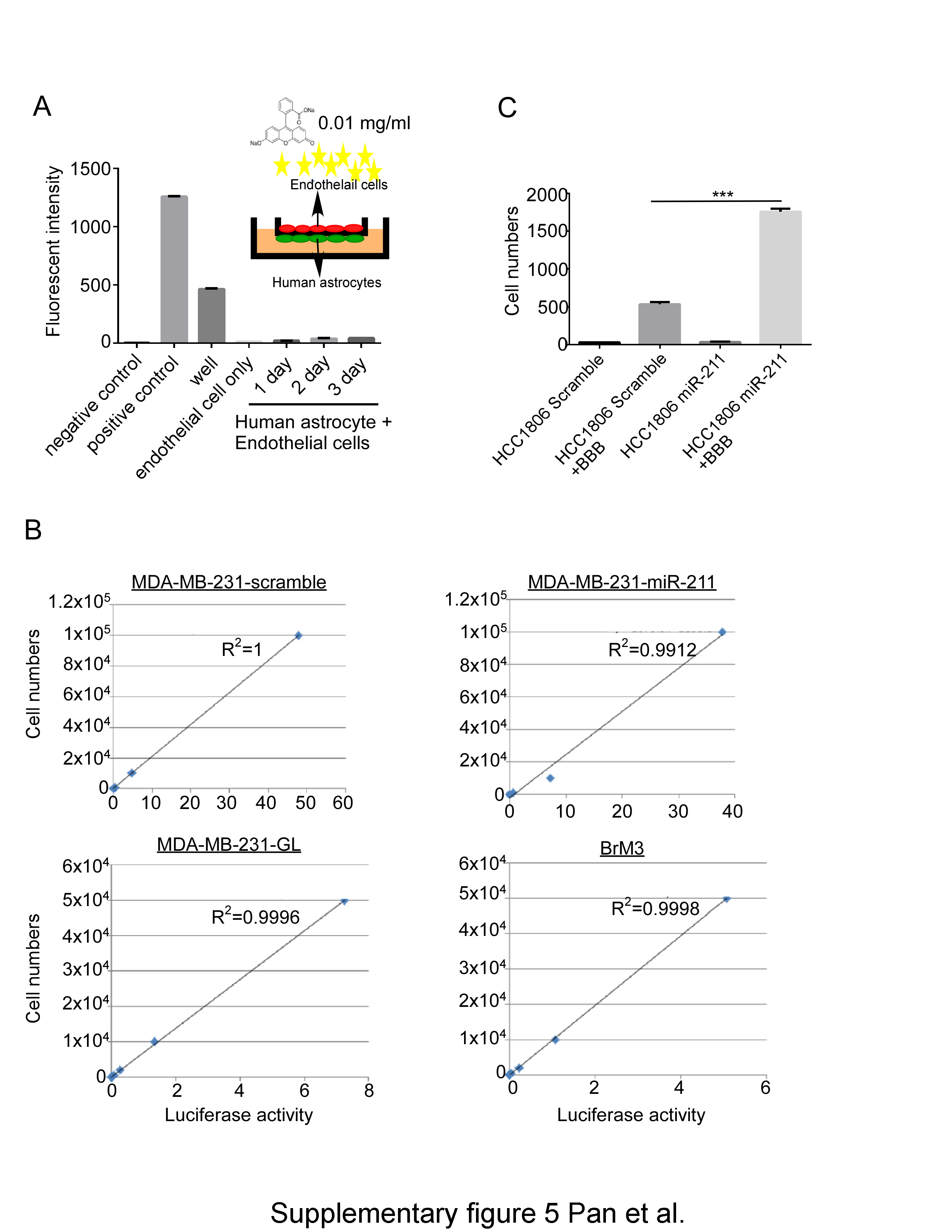

Supplement: Supplementary file 6 — Figure S5 [file 41388_2021_1654_MOESM6_ESM.jpg]

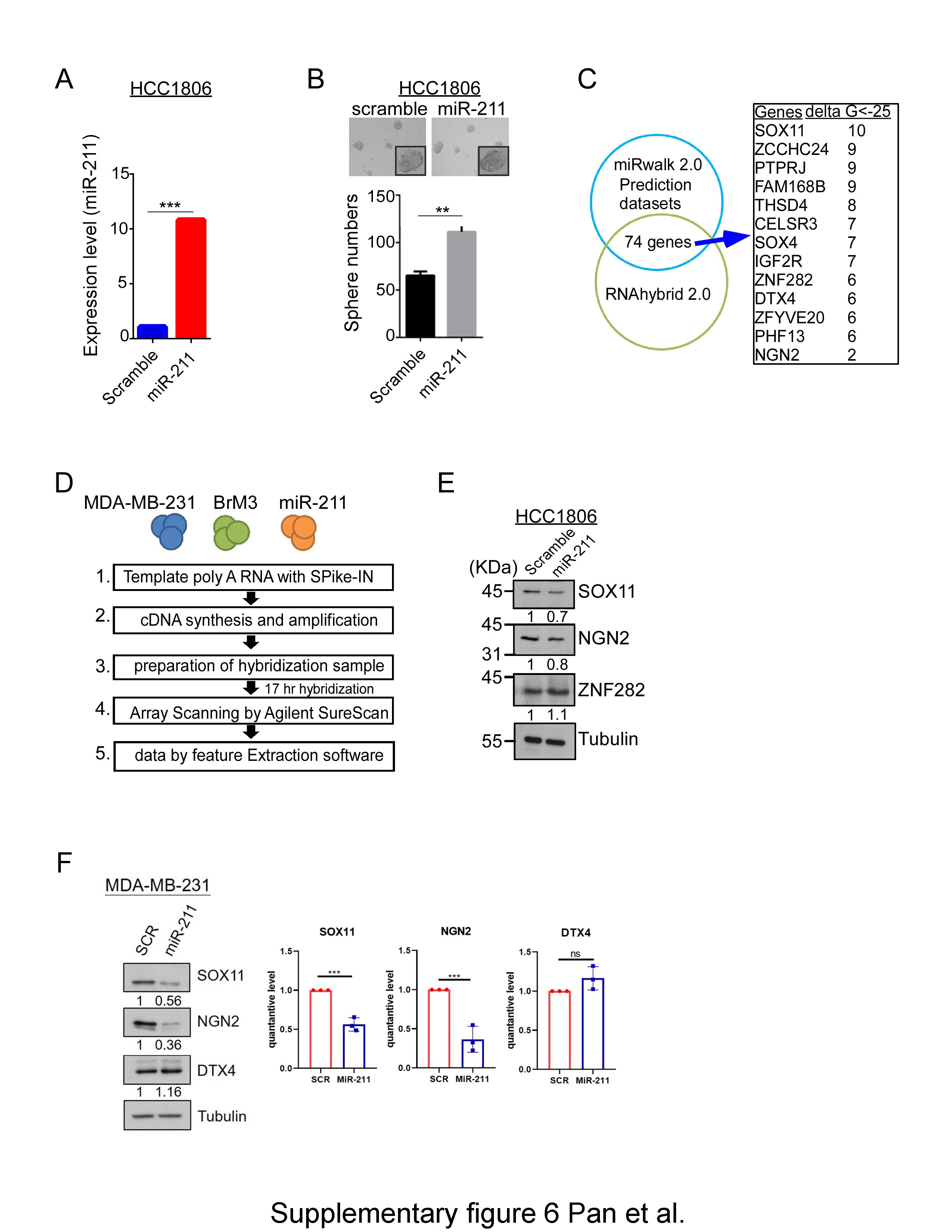

Supplement: Supplementary file 7 — Figure S6 [file 41388_2021_1654_MOESM7_ESM.jpg]

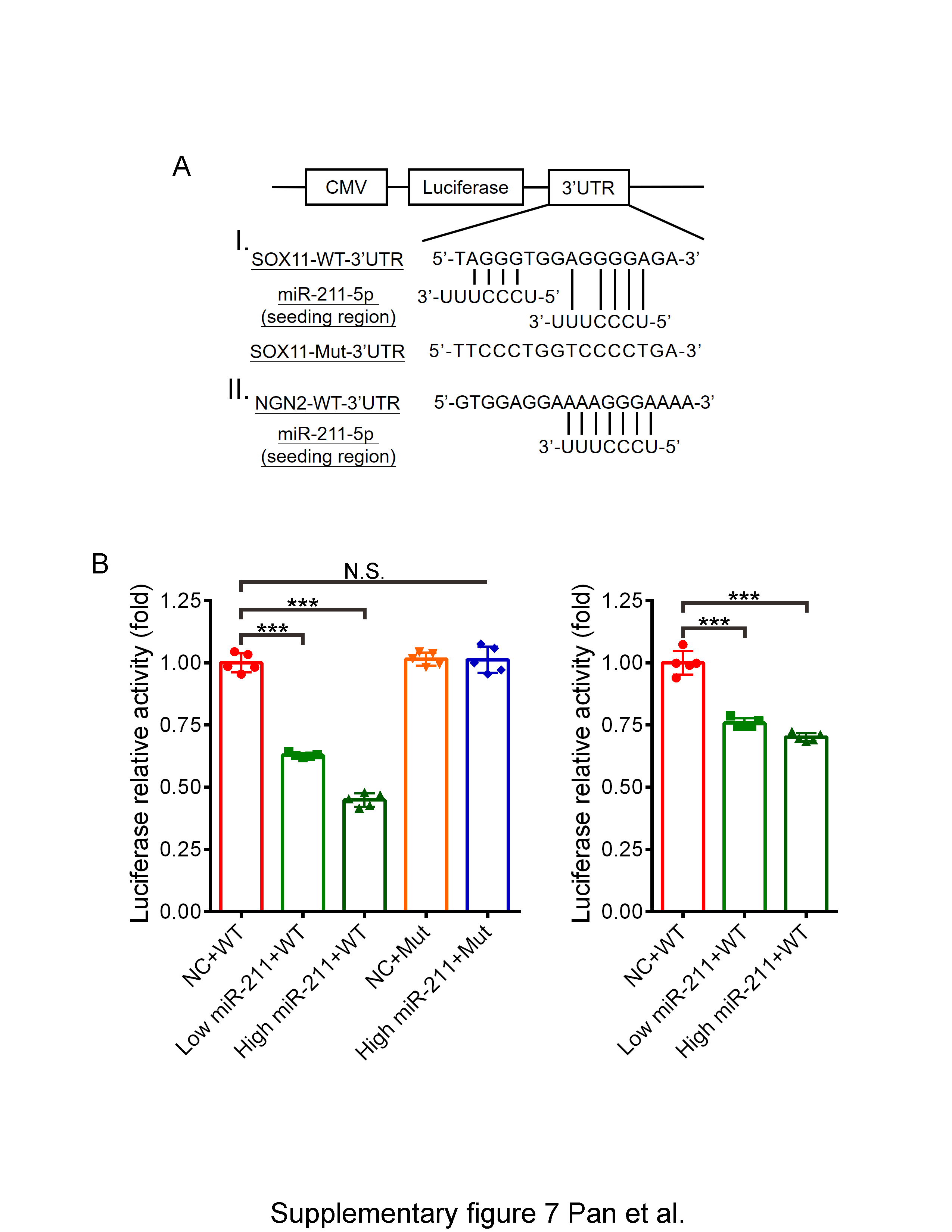

Supplement: Supplementary file 8 — Figure S7 [file 41388_2021_1654_MOESM8_ESM.jpg]

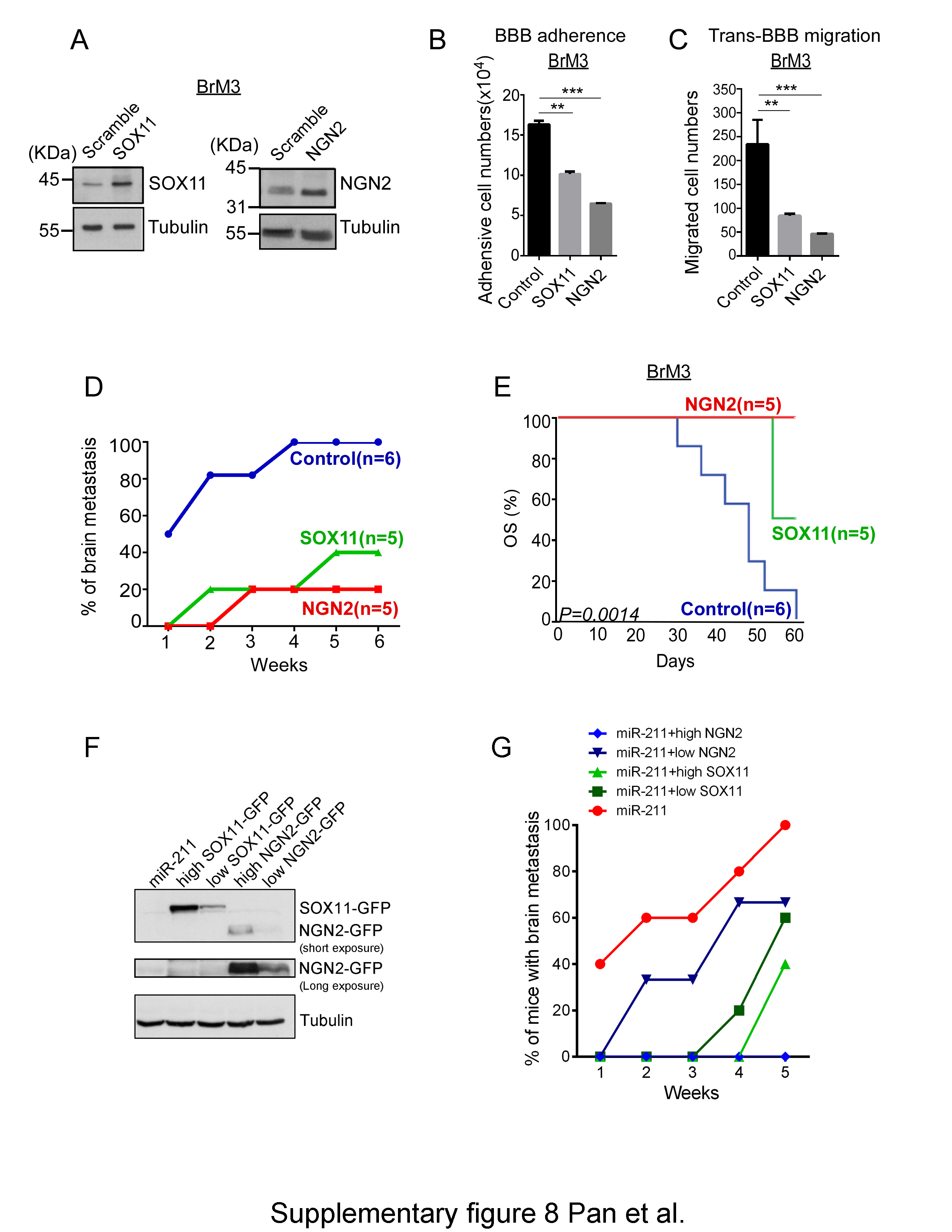

Supplement: Supplementary file 9 — Figure S8 [file 41388_2021_1654_MOESM9_ESM.jpg]
